# Calcium Signaling Dynamics in Vascular Cells and Their Dysregulation in Vascular Disease

**DOI:** 10.3390/biom15060892

**Published:** 2025-06-18

**Authors:** Chang Dai, Raouf A. Khalil

**Affiliations:** Vascular Surgery Research Laboratories, Division of Vascular and Endovascular Surgery, Brigham and Women’s Hospital, Harvard Medical School, Boston, MA 02115, USA; daichang@gdph.org.cn

**Keywords:** channels, contraction, endothelium, extracellular matrix, hypertension, smooth muscle

## Abstract

Calcium (Ca^2+^) signaling is a fundamental regulatory mechanism controlling essential processes in the endothelium, vascular smooth muscle cells (VSMCs), and the extracellular matrix (ECM), including maintaining the endothelial barrier, modulation of vascular tone, and vascular remodeling. Cytosolic free Ca^2+^ concentration is tightly regulated by a balance between Ca^2+^ mobilization mechanisms, including Ca^2+^ release from the intracellular stores in the sarcoplasmic/endoplasmic reticulum and Ca^2+^ entry via voltage-dependent, transient-receptor potential, and store-operated Ca^2+^ channels, and Ca^2+^ elimination pathways including Ca^2+^ extrusion by the plasma membrane Ca^2+^-ATPase and Na^+^/Ca^2+^ exchanger and Ca^2+^ re-uptake by the sarco(endo)plasmic reticulum Ca^2+^-ATPase and the mitochondria. Some cell membranes/organelles are multifunctional and have both Ca^2+^ mobilization and Ca^2+^ removal pathways. Also, the individual Ca^2+^ handling pathways could be integrated to function in a regenerative, capacitative, cooperative, bidirectional, or reciprocal feed-forward or feed-back manner. Disruption of these pathways causes dysregulation of the Ca^2+^ signaling dynamics and leads to pathological cardiovascular conditions such as hypertension, coronary artery disease, atherosclerosis, and vascular calcification. In the endothelium, dysregulated Ca^2+^ signaling impairs nitric oxide production, reduces vasodilatory capacity, and increases vascular permeability. In VSMCs, Ca^2+^-dependent phosphorylation of the myosin light chain and Ca^2+^ sensitization by protein kinase-C (PKC) and Rho-kinase (ROCK) increase vascular tone and could lead to increased blood pressure and hypertension. Ca^2+^ activation of matrix metalloproteinases causes collagen/elastin imbalance and promotes vascular remodeling. Ca^2+^-dependent immune cell activation, leukocyte infiltration, and cholesterol accumulation by macrophages promote foam cell formation and atherosclerotic plaque progression. Chronic increases in VSMCs Ca^2+^ promote phenotypic switching to mesenchymal cells and osteogenic transformation and thereby accelerate vascular calcification and plaque instability. Emerging therapeutic strategies targeting these Ca^2+^-dependent mechanisms, including Ca^2+^ channel blockers and PKC and ROCK inhibitors, hold promise for restoring Ca^2+^ homeostasis and mitigating vascular disease progression.

## 1. Introduction

Calcium is one of the most abundant and essential elements in the body, accounting for 1–2% of total body weight, with the vast majority, ~99%, stored in the bones and teeth as hydroxyapatite crystals [1]. The remaining small fraction of calcium, ~1%, is found in the serum, extracellular fluid, and different cellular compartments, where it is involved in a wide range of physiological and regulatory activities [2]. Serum calcium is usually measured by an autoanalyzer using colorimetry and ion-selective electrodes or by atomic absorption spectrophotometry for measurements requiring high precision. In the bloodstream, serum total calcium exists in two forms: free ionized Ca^2+^, which is biologically active, and Ca^2+^ bound to proteins or complexed with anions, which together maintain a tightly regulated serum concentration of 8.6–10.2 mg/dL total calcium and 4.6 to 5.2 mg/dL or 2.1–2.6 mmol/L free ionized Ca^2+^ [3]. The serum total calcium and free ionized Ca^2+^ levels are maintained by a balance between Ca^2+^ absorption, metabolism, binding, deposition, and excretion processes that are regulated by vitamin D, parathormone, and various gastrointestinal absorption and renal excretion mechanisms.

Ca^2+^ is an indispensable second messenger in almost all types of cells and biological systems. Cytosolic free Ca^2+^ concentration ([Ca^2+^]_c_) was initially assessed in large-sized cells by injecting the metallochromic indicator antipyralzo III or arsenazo III [4] or the bioluminescent protein aequorin [5,6] or using a Ca^2+^-sensitive microelectrode [7]. These microinjection and impalement techniques are very challenging in small vascular cells, including endothelial cells (ECs) and vascular smooth muscle cells (VSMCs). One way to circumvent these technical difficulties has been to administer aequorin in vascular preparations by transient membrane permeabilization [8]. Also, fluorescent Ca^2+^ indicators including quin-2, fura-2, indo-1, and Fluo-3 and -4 were developed to facilitate measurement of [Ca^2+^]_c_ in numerous cell types, including vascular cells [9,10,11,12,13,14,15]. Living cells are incubated in the presence of the Ca^2+^ indicator acetoxymethyl ester, which can diffuse through the surface membrane to the cytosol, where it is met by intracellular esterases and hydrolyzed into the hydrophilic free acid that is trapped inside the cell and changes its fluorescence properties upon binding to free ionized Ca^2+^. These various techniques have registered a physiological [Ca^2+^]_c_ in the range of 0.1 to 1 μmol/L in most cell types.

The role of Ca^2+^ is particularly critical in cardiovascular physiology, where it functions not only as a fine-tuning regulator of gene expression, metabolism, and cell survival but also as a modulator of vascular tone and a driver of vascular contraction and remodeling. Importantly, the Ca^2+^ concentration gradient across the cell membrane is striking. While extracellular Ca^2+^ concentration is typically maintained at 1–2 mmol/L, [Ca^2+^]_c_ is maintained at a dramatically lower level, ~100 nmol/L (0.1 µmol/L), in resting cells. Also, although [Ca^2+^]_c_ is elevated in activated cells, it does not exceed 1–2 µmol/L [16,17]. This creates a large, ~1000-fold concentration gradient across the plasma membrane that continuously drives Ca^2+^ from the extracellular space into the cell microenvironment, where it could be fatal. Fortunately, the cell is equipped with precise and adaptable pathways that maintain cell stability, allow enough Ca^2+^ to activate the cell, and safeguard against excessive increases in [Ca^2+^]_c_. This is achieved through a balance between Ca^2+^ mobilization mechanisms including Ca^2+^ release from the intracellular Ca^2+^ storage sites and Ca^2+^ influx via plasmalemmal channels, and Ca^2+^ elimination mechanisms including Ca^2+^ extrusion pumps and exchangers, Ca^2+^ uptake to replenish intracellular stores, and Ca^2+^ sequestration in designated Ca^2+^ depots. This delicate balance between Ca^2+^ entry, storage, and removal ensures that [Ca^2+^]_c_ remains tightly controlled, allowing for precise regulation of cellular functions while preventing Ca^2+^ overload and cytotoxicity. Intricate regulation of Ca^2+^ movement through these different pathways is crucial for maintaining cellular homeostasis. Disruption in these finely tuned Ca^2+^ handling mechanisms can lead to cardiovascular pathology and various vascular disorders. Understanding the mechanisms of Ca^2+^ signaling and regulatory pathways would be useful to elucidate the pathophysiological basis of vascular disease and help in the development of therapeutic strategies to restore Ca^2+^ homeostasis and prevent disease progression.

In this review we use data from scientific reports published in PubMed and additional data from our research group to offer insights into the Ca^2+^ signaling mechanisms in vascular cells and the dysregulation of Ca^2+^ dynamics in vascular disease. We will first describe the mechanisms controlling [Ca^2+^]_c_ under physiological resting conditions and in activated vascular cells. We will describe how the Ca^2+^ dynamics in certain membranes/organelles have evolved to be multifunctional (e.g., plasma membrane Ca^2+^ entry channels vs. opposing plasma membrane Ca^2+^-ATPase (PMCA) pump, sarcoplasmic reticulum (SR)/endoplasmic reticulum (ER) Ca^2+^ release vs. sarco(endo)plasmic reticulum Ca^2+^-ATPase (SERCA) uptake, and mitochondrial rescue Ca^2+^ sequestration vs. slow Ca^2+^ release), regenerative (SR/ER Ca^2+^-induced Ca^2+^ release, CICR), capacitative (store-operated Ca^2+^ entry), cooperative (superficial Ca^2+^ gradient/buffer barrier involving Ca^2+^ uptake by SERCA followed by extrusion via PMCA or Na^+^/Ca^2+^ exchanger (NCX)), bidirectional (NCX forward-mode Ca^2+^ extrusion and reverse-mode Ca^2+^ entry), and reciprocal feed-forward or feed-back mechanisms (Ca^2+^ stimulation of protein kinase-C (PKC) and PKC-mediated modulation of Ca^2+^ channels and activation of PMCA). We will then describe the role of Ca^2+^ in regulating various cellular functions and processes in ECs, VSMCs, and ECM. We will follow with the pathological changes in Ca^2+^ regulatory mechanisms underlying vascular diseases such as hypertension (HTN), age-related arterial stiffness, atherosclerosis, and coronary artery disease (CAD). We will finally discuss how understanding the Ca^2+^ signaling dynamics could help to elucidate the pathogenesis of vascular disorders and suggest potential approaches for targeted intervention and management of cardiovascular disease [18,19,20,21].

## 2. Ca^2+^ Mobilization Pathways

In 1883, Ringer’s experiments on the isolated heart showed that the presence of Ca^2+^ was critical for the cardiac muscle to perform its function and maintain its activity [22]. Heilbrunn and coworkers also confirmed the important role of intracellular Ca^2+^ in the muscle contractile response [23]. Intracellular Ca^2+^ is derived from Ca^2+^ release from the intracellular storage sites and Ca^2+^ entry from the extracellular environment. Electrophysiological and voltage-clamp studies supported the Ca^2+^ channel as a physiologically distinct entity with a critical role in excitation–contraction coupling [24,25,26]. Also, patch-clamp and tight-seal measurements allowed the detection of Ca^2+^ passage through a single channel in cardiomyocytes [27]. Electrophysiological research has advanced steadily and led to the finding of several forms of Ca^2+^ channels with distinct molecular structure, biophysical characteristics, functions, activators, and inhibitors.

## 3. Ca^2+^ Release from Intracellular Stores

In the presence of extracellular Ca^2+^, vasoconstrictor agonists produce an initial fast contractile response followed by a sustained contraction. Ca^2+^ release from the intracellular stores is an important initiating event in agonist-evoked contraction of VSMCs. In blood vessels incubated in Ca^2+^-free medium, vasoconstrictors mainly elicit a transient contractile response [18,28]. Also, in blood vessels treated with Ca^2+^ channel antagonists, vasoconstrictors fail to produce sustained contraction and ^45^Ca^2+^ influx, but a small transient contractile response can still be detected [18,29,30]. Furthermore, in vascular preparations loaded with radioactive ^45^Ca^2+^ then incubated in a Ca^2+^-free medium, vasoconstrictor agonists stimulate Ca^2+^ efflux [31].

Electron microscopy of SMC ultrastructure showed entities akin to SR that can take Ca^2+^ from ambient media that contain micromolar levels of Ca^2+^ [32]. Structurally, the SR consists of interconnected tubules or flattened cisternae [32,33] that account for 1.5–7.5% of SMC volume [34]. The ratio of SR volume/total SMC volume has been correlated with the strength of cell contraction, particularly in the absence of external Ca^2+^, such that the greater the ratio, the stronger the SMC contraction. For instance, large arteries, such as the rabbit aorta and pulmonary artery, have a large SR volume and therefore a large contraction in the absence of extracellular Ca^2+^. On the other hand, phasic SMC tissues, such as guinea pig taenia coli and rabbit mesenteric veins, have a smaller SR, ~1.5 to 2.5% of the total cell volume [35], and therefore elicit a miniscule contractile response in Ca^2+^-free solution.

Cell fractionation techniques have managed to isolate the microsomal SR fraction from different cells. Isolated SMC SR microsomes accumulate radioactive ^45^Ca^2+^ and release it upon stimulation by Ca^2+^-mobilizing drugs, including caffeine and ryanodine. Ca^2+^ release channels were also detected in isolated SR vesicles reconstituted in planar lipid bilayer [36]. Because of the loss of critical cellular factors during the fractionation and purification of SR vesicles, SMC preparations chemically permeabilized by saponin or α-toxin have been used to assess the Ca^2+^ release mechanism under more physiological settings [33,37,38,39,40]. SR release of Ca^2+^ is stimulated by inositol 1,4,5-trisphosphate (IP_3_) and the activation of the IP_3_ receptor (IP_3_R) or by Ca^2+^-induced Ca^2+^ release (CICR) and stimulation of the ryanodine receptor (RyR).

### 3.1. IP_3_-Induced Ca^2+^ Mobilization from SR

**Figure 1:**
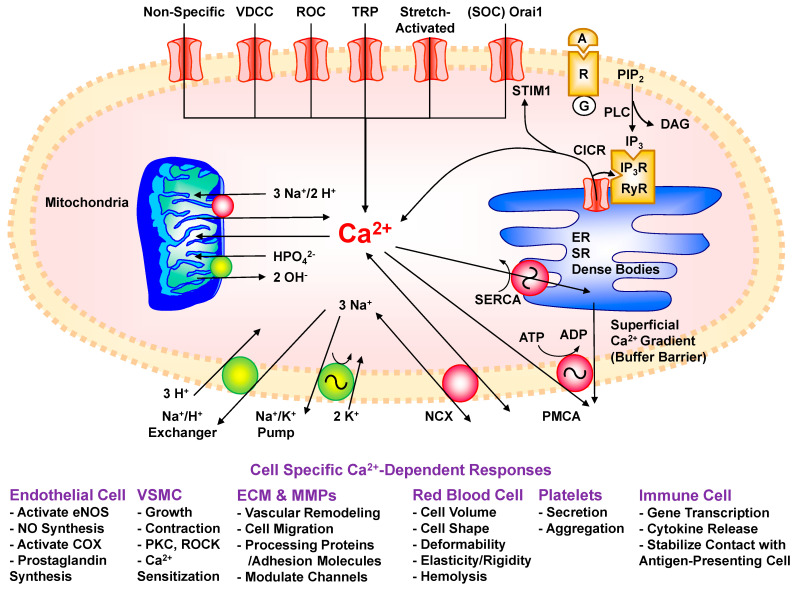
Regulation of [Ca^2+^]_c_ in vascular cells and Ca^2+^-dependent cellular responses. A vasoconstrictor agonist (A) interacts with its specific receptor (R) and induces Ca^2+^ mobilization from the endoplasmic reticulum (ER), sarcoplasmic reticulum (SR) or dense bodies in response to increases in 1,4,5-inositol trisphosphate (IP_3_) production, initial release of Ca^2+^, and subsequent activation of Ca^2+^-induced Ca^2+^ release (CICR). Vasoconstrictor agonists also enhance Ca^2+^ entry via nonspecific Ca^2+^ leak pathway and various voltage-dependent (VDCC), receptor-operated (ROC), transient receptor potential (TRP), and stretch-activated channels. Depleted intracellular Ca^2+^ storage sites in ER/SR trigger the release of stromal interaction molecule-1 (STIM1) and stimulation of Ca^2+^ entry through Orai1 store-operated Ca^2+^ channels (SOCs). Increased intracellular Ca^2+^ is taken up by ER/SR Ca^2+^-ATPase (SERCA) or removed to the extracellular space via the plasma membrane Ca^2+^-ATPase (PMCA) or Na^+^/Ca^2+^ exchanger (NCX), with the resulting increase in intracellular Na^+^ being removed through the Na^+^/K^+^ pump or Na^+^/H^+^ exchanger. When increases in [Ca^2+^]_c_ are dangerously high and become pathological, the mitochondria come to the rescue to take up excess Ca^2+^ and maintain homeostasis. As the mitochondria take up Ca^2+^, they also take up HPO_4_^2−^ via HPO_4_^2−^:2OH^−^ exchange to form calcium phosphate. When the conditions become more favorable for the SERCA, PMCA, and NCX to maintain [Ca^2+^]_c_, the mitochondria slowly release Ca^2+^ via Ca^2+^ efflux and associated electrogenic Ca^2+^:3Na^+^ or electroneutral Ca^2+^:2H^+^ antiporter. Increases in [Ca^2+^]_c_ trigger a range of responses in ECs, VSMCs, ECM, red blood cells, platelets, and immune cells. PIP_2_, phosphatidylinositol 4,5-bisphosphate; PLC, phospholipase C; DAG, diacylglycerol.

Neurotransmitters, hormones, and other agonists activate G-protein-coupled receptors (GPCRs) and stimulate phospholipase C (PLC), which catalyzes the breakdown of plasmalemmal phosphatidylinositol 4,5-bisphosphate (PIP_2_) into IP_3_ and 1,2-diacylglycerol (DAG) [41]. IP_3_ is hydrophilic and diffuses through the cytosol to trigger Ca^2+^ release from ER/SR [42,43,44,45] (Figure 1). Conversely, DAG is hydrophobic and remains in the plasma membrane to promote PKC translocation and activation [20,46,47]. IP_3_ binds to IP_3_R in SR and activates Ca^2+^ release channels. In saponin-permeabilized SMCs, IP_3_ at a half-maximal effective concentration (EC_50_) of ~1 μmol/L causes rapid and large Ca^2+^ release from SR and a transient SMC contraction [42,44,48]. As a second messenger, IP_3_ is rapidly inactivated by endogenous 5-phosphatase into inositol 1,4-bisphosphate (IP_2_) [41,49]. IP_3_-induced Ca^2+^ release is modulated by phosphorylation and other regulatory proteins, depending on the cellular needs and stress responses. Because heparin has an electronegative charge, it can compete with IP_3_ to block IP_3_R and IP_3_-mediated Ca^2+^ release [50]. Also, mice with genetically deleted IP_3_R show reduced aortic contraction to the vasoconstrictor agonists phenylephrine, endothelin-1 (ET-1), U46619, and serotonin, suppressed U46619-induced myosin light chain (MLC-20) phosphorylation, and attenuated pressor response to angiotensin II (Ang II) infusion, providing further evidence for a role of IP_3_R-induced Ca^2+^ release in mediating VSMC contraction and regulating blood pressure (BP) [51].

### 3.2. Ca^2+^-Induced Ca^2+^ Release (CICR)

In permeabilized skeletal [33,52], cardiac [53], and vascular preparations [37], application of small Ca^2+^ concentrations causes further Ca^2+^ release or CICR from SR. CICR is a regenerative cellular event accelerated by Ca^2+^-mobilizing agents, including caffeine and ryanodine, through activation of the ryanodine receptor (RyR) [33]. CICR provides rapid, high-amplitude Ca^2+^ signal for activation of contractile proteins and augmenting muscle contraction. A threshold 3 µmol/L increase in [Ca^2+^]_c_ near SR is sufficient to activate RyR and trigger CICR. In cardiac muscle, Ca^2+^ influx through L-type voltage-dependent Ca^2+^ channels (VDCCs) activates cardiac RyR2, triggering CICR. In contrast, in skeletal muscle, excitation–contraction coupling primarily depends on a depolarization-induced conformational change in modified L-type VDCCs, also known as dihydropyridine receptors (DHPRs), which serve as pure voltage sensors and are mechanically and directly coupled to RyR1. Because DHPRs have negligible permeability for Ca^2+^, it is assumed that there is no considerable Ca^2+^ influx through DHPRs in skeletal muscle that could contribute to CICR [54,55]. RyR and CICR are modulated by phosphorylation and redox factors and are potentiated by 3′,5′-cyclic adenosine monophosphate (cAMP) and reduced by Mg^2+^ or procaine [37]. In many cell types, both IP_3_-induced Ca^2+^ release and CICR act synergistically to amplify the Ca^2+^ signal. It is thought that an initial IP_3_-induced Ca^2+^ release raises [Ca^2+^]_c_ in the vicinity of SR above the 3 µmol/L threshold and triggers further amplification of Ca^2+^ release through CICR [56]. In support, Ca^2+^ enhances IP_3_-induced Ca^2+^ release from SR in permeabilized guinea pig taenia caeci SMCs [48]. Also, studies utilizing calsequestrin-targeted Ca^2+^ indicator showed that ET-1-induced Ca^2+^ release waves from VSMC SR. A transient increase in SR Ca^2+^ level was detected both at the wave initiation site, immediately prior to the beginning of regenerative Ca^2+^ release, and at the progressing wave front during propagation, supporting a role of SR luminal Ca^2+^ in IP_3_R stimulation during vasoconstrictor-induced Ca^2+^ waves, likely through regenerative CICR [57]. Studies have also suggested that both IP_3_Rs and RyRs are ligand-gated ion channels and thus exist as single functional proteins [58].

## 4. Ca^2+^ Entry from the Extracellular Environment

Ca^2+^ can enter VSMCs via multiple pathways, including non-selective Ca^2+^ leak pathways and more specific voltage-dependent, receptor-operated, transient receptor potential (TRP), store-operated, and stretch-activated Ca^2+^ channels (Figure 1).

### 4.1. Ca^2+^ Leak and Non-Specific Channels

The high Ca^2+^ electrochemical and concentration gradient across the plasma membrane drives continuous Ca^2+^ entry into VSMCs via Ca^2+^ leak channels. The Ca^2+^ leak passageway is lined up with carboxylate and phosphate moieties, partially inhibited at low pH, and can be reduced by ~66% using cobalt or lanthanum [18]. Initially, the Ca^2+^ leak was believed to be a non-specific ion passage across the surface membrane but was later recognized as a divalent cation-selective channel, which displays occasional spontaneous openings [59] and opens at holding potentials below the threshold for VDCC activation, with a greater conductance than the adenosine triphosphate (ATP)-sensitive and receptor-operated Ca^2+^ channel (ROC). Using ^45^Ca^2+^, the Ca^2+^ leak in rabbit aorta was estimated at ~14 μmol/kg/min [28], but such a high Ca^2+^ entry does not trigger a vascular contractile response, as it is counterbalanced by Ca^2+^ re-uptake via SERCA and Ca^2+^ removal via PMCA. However, Ca^2+^ leak could contribute to VSM contraction under conditions associated with compromised Ca^2+^ removal mechanisms or increased Ca^2+^ sensitivity of contractile myofilaments.

### 4.2. Voltage-Dependent Ca^2+^ Channels (VDCCs)

Most blood vessels require extracellular Ca^2+^ to maintain contraction. In Ca^2+^-free medium, rabbit aortic segments fail to elicit contraction to membrane depolarization by high KCl, and they show marked decrease in norepinephrine-induced contraction. In contrast, in Ca^2+^-containing medium high KCl stimulates ^45^Ca^2+^ influx and vascular contraction, and these responses are inhibited by Ca^2+^ channel blockers such as dihydropyridines [18,29]. Also, Ca^2+^ channel agonists such as Bay-K8644 stimulate Ca^2+^ influx and vascular contraction, suggesting a distinct Ca^2+^ entry passage triggered by plasma membrane depolarization that is designated as VDCC [60,61,62]. VDCCs are transmembrane proteins classified into L-, T-, N-, P/Q-, and R-type, each with distinct tissue distributions and physiological roles. In neurons, L- and T-type VDCCs mediate maintained Ca^2+^ influx and neuronal excitability [63,64]. N-, P/Q-, and R-type VDCCs are predominantly localized at presynaptic terminals, where they cause localized increases [Ca^2+^]_c_ and facilitate neurotransmitter vesicle fusion, exocytosis, and release [65,66]. In cardiovascular cells, voltage-dependent Ca^2+^ current has two components. The L-type or long-lasting component is activated by large depolarization stimuli and inactivates slowly, while the T-type or transient component is activated by small depolarizations and inactivates rapidly [67]. Both L- and T-type Ca^2+^ currents are inhibited by cadmium, cobalt or lanthanum [68,69,70,71], but have distinct sensitivities to dihydropyridines. The L-type current is blocked by nifedipine, nimodipine, nisoldipine, and nitrendipine and enhanced by Bay-K8644 and Bay-R5417, while the T-type current is not affected [67,68,70]. Also, physiological agonists do not often stimulate voltage-activated Ca^2+^ current [67,68,70]. However, in single rabbit ear artery VSMCs, norepinephrine, through a non-α non-β adrenergic receptor, activates the L-type but not the T-type current [72]. Also, in rabbit mesenteric artery VSMCs, norepinephrine enhances VDCCs’ open probability [62].

Molecular studies showed 65% amino acid sequence homology between the rabbit lung L-type Ca_V_1.2 channel (LTCC) and its corresponding channel in skeletal muscle [73]. LTCC is composed of a pore-forming α_1c_ subunit and auxiliary β, α_2_δ, and γ subunits that regulate its activity [74]. The α_1c_ subunit comprises a voltage sensor, a gating module, and the Ca^2+^-permeable pore, which contains four homologous domains, I, II, III, IV, with each domain having six transmembrane segments, S1–S6, an intracellular NH_2_-terminus, and a COOH-terminus. The channel pore resides in the S5 and S6 segments, and the Ca^2+^ selectivity is determined by two glutamates at the pore loop, while the voltage sensor resides in the S1–S4 segments and can rotate to allow opening of the channel pore [74,75]. VSMC Ca_V_1.2 activity is augmented at highly depolarized membrane potential (−45 to −36 mV) and during large increases in luminal vascular pressure [76].

T-type Ca^2+^ channel (TTCC) was first discovered in guinea pig cardiomyocytes and shows a transient ~8 pS conductance when Ba^2+^ was used as the charge carrier. T-type Ca^2+^ current can be activated at ~−30 mV membrane potential and is not blocked by nanomolar concentrations of dihydropyridines. TTCCs have Ca_V_3.1, Ca_V_3.2, and Ca_V_3.3 isoforms. Ca_V_3.1 and Ca_V_3.3 play a role in mediating myogenic tone at low, 20–40 mmHg luminal vascular pressure and ~−60 to −50 mV membrane potential [77]. Ca_V_3.2 functions as a negative feed-back regulator of pressure-mediated vascular tone through modulation of the activity of large conductance Ca^2+^-activated K^+^ channel (BK_Ca_) [78].

Ca^2+^-dependent inactivation of LTCC is an important feed-back mechanism that involves binding of Ca^2+^/calmodulin (CaM) to the pore-forming α_1c_ subunit at the C-terminus and thereby limiting the elevation of [Ca^2+^]_c_. The T-type Ca_v_3.3 channel is also regulated by the Ca^2+^/CaM binding interaction to its carboxyl terminus, providing an additional negative feed-back mechanism that prevents excess Ca^2+^ passage via VDCCs [79].

### 4.3. Receptor-Operated Ca^2+^ Channels (ROCs)

Vasoconstrictors can stimulate Ca^2+^ channels other than VDCCs. In rabbit aorta maximally stimulated by high KCl-mediated membrane depolarization, norepinephrine produces further contraction. Also, ^45^Ca^2+^ influx measured during combined activation with the maximum concentrations of norepinephrine and KCl is equivalent to the sum of ^45^Ca^2+^ influx activated by each stimulus separately, suggesting additive effects of norepinephrine and KCl on Ca^2+^ entry [18,80]. Additionally, ^45^Ca^2+^ influx activated by maximal concentrations of a Ca^2+^ channel agonist such as Bay-K8644 can be additive during combined stimulation with norepinephrine but not KCl. Furthermore, dihydropyridines block high KCl but not norepinephrine-evoked VSMC contractile response or Ca^2+^ entry [80]. These observations suggest that physiological agonists stimulate specific receptors and activate ROCs that are different from VDCCs [60,61]. In support, electrophysiological studies demonstrated that ATP activated a different Ca^2+^ current in rabbit ear artery VSMCs [59]. The ATP-activated channel is 3:1 more selective to Ca^2+^ over Na^+^ and, in contrast with VDCCs, is not blocked by cadmium or nifedipine, opens at a higher negative potential, and has ~5 pS unitary conductance when using 110 mmol/L Ba^2+^ or Ca^2+^ as the charge carrier. Importantly, the ATP-sensitive channel is not stimulated if ATP is applied to the outside of the cell-attached patch pipette, indicating that it requires direct activation by an ATP-sensitive surface receptor rather than ATP-induced production of a second messenger [81]. ROCs are now considered a subtype of the transient receptor potential (TRP) family of channels [82].

### 4.4. Transient Receptor Potential (TRP) Channels

TRP channels comprise a family of cation channels that are encoded by 28 genes and include six subfamilies in mammals: TRPC (canonical), TRPV (vanilloid), TRPM (melastatin), TRPP (polycystin), TRPA (ankyrin), and TRPML (mucolipin). TRPN (NO-mechano-potential, NOMP) has been identified in worms, fruit flies, and zebrafish [83]. TRP channels contribute to the regulation of VSMC membrane potential, contraction, and myogenic tone. Also, certain TRP channels play a role in the mechanosensitivity of small resistance microvessels. The majority of TRP channels show high Ca^2+^ permeability, whereas TRPM4 and TRPM5 can be activated by Ca^2+^ but show less permeability to Ca^2+^ [84].

Vasoconstrictor agents activate Ca^2+^ influx via non-selective cation channels belonging to the TRPC subfamily. TRPCs can be activated secondary to receptor stimulation (ROCs) or following depletion of the intracellular Ca^2+^ storage sites and activation of capacitative Ca^2+^ influx through store-operated channels (SOCs). TRPC channels simultaneously allow Ca^2+^ and Na^+^ influx, leading to depolarization of the surface membrane and elevation of [Ca^2+^]_c_ [82,85]. All TRPCs, except TRPC2 and TRPC7, have been identified in VSMCs at different levels in various vascular beds. TRPC1 and TRPC6 are very abundant in VSMCs. TRPC4 is expressed to a lesser extent in rat aorta and mesenteric, renal, and cerebral arteries and are not detected in the caudal artery. TRPC3 is expressed to a greater extent in rat renal, caudal, and cerebral arteries compared to the aorta. TRPC5 is expressed at low levels in rat aorta and renal arteries but is not found in the mesenteric arteries [82].

### 4.5. Store-Operated Channels (SOCs)

Agonist–receptor interaction is associated with an initial Ca^2+^ mobilization from the intracellular storage sites, followed by sustained Ca^2+^ influx from the extracellular environment. Depletion of Ca^2+^ storage sites in ER or SR could function as a capacitor to stimulate “capacitative” or “store-operated” Ca^2+^ influx [86,87,88]. There is accumulating evidence of Ca^2+^ entry through SOCs [89,90]. Cyclopiazonic acid and thapsigargin inhibit SERCA and deplete SR Ca^2+^ storage sites without stimulating GPCRs, thereby allowing differentiation of Ca^2+^ entry via SOCs compared to ROCs. In VSMCs, application of thapsigargin depletes SR Ca^2+^ and stimulates Ca^2+^ entry that is not associated with IP_3_ production and is not inhibited by blockers of L-type VDCCs such as nicardipine [91]. However, SERCA inhibitor-induced Ca^2+^ entry depends on the presence of Ca^2+^ in the extracellular space and is sufficient to sustain vascular contraction [92,93].

Canonical TRPCs such as TRPC1 and TRPC5 are important mediators of store-operated Ca^2+^ entry in VSMCs [94,95,96,97]. TRPC1 is linked to TRPP2 (polycystin-2) [98], and TRPC5 is a major component of SOCs [97]. TRPC3, TRPC4, and TRPC7 mediate store-operated Ca^2+^ entry mainly in nonvascular cells [99,100]. In airway SMCs, IP_3_-induced depletion of Ca^2+^ stores leads to the activation of plasmalemmal TRPC3 and subsequent Ca^2+^ influx [101], supporting the presence of a store-operated Ca^2+^ entry pathway.

In VSMCs from mouse aorta, depletion of intracellular Ca^2+^ storage sites may stimulate the production of a Ca^2+^ influx factor (CIF), which in turn activates SOCs [102]. Studies in inside-out SMC membrane patches showed that a CIF extract from yeast could mediate the activation of a 3-pS Ca^2+^ channel following SR Ca^2+^ depletion by thapsigargin or 1,2-bis(o-aminophenoxy)ethane-N,N,N′,N′-tetraacetic acid (BAPTA) [103]. CIF was isolated in a partially pure stable form, but its structural characteristics were not well-defined [104,105]. Stromal-interacting molecule 1 (STIM1) is a membrane-spanning protein that senses Ca^2+^ levels inside the storage sites and activates TRPC1 and store-operated Ca^2+^ entry [106,107]. Orai1 may represent the pore-forming subunit of SOCs [108]. The interaction of STIM1 with Orai1 leads to SOC gain of function [109,110]. As STIM1 senses depletion of intracellular Ca^2+^ stores by physiological stimuli, it relocates to surface membrane junctions within ER/SR, then interacts with and activates Orai1 channels in the plasma membrane. Septins are cytoskeletal proteins that self-associate, polymerize, and bind to the surface membrane [111] and have been suggested as potential coordinators of store-operated Ca^2+^ entry [112]. Septin filaments rearrange locally with PIP_2_ at the ER-surface membrane junction in parallel with the formation of STIM1–Orai1 clusters, suggesting that they may facilitate STIM1 targeting, Orai1 recruitment, and STIM1–Orai1 interaction to maintain store-operated Ca^2+^ entry [113]. The formation of STIM1–Orai1 clusters and maintained Ca^2+^ entry activate Ca^2+^-dependent transcription factors such as nuclear factor of activated T cells 1 (NFAT1) and c-Fos, which regulate the synthesis of different proteins/enzymes for various cellular responses [114].

### 4.6. Stretch-Activated Ca^2+^ Channels

“Autoregulation” of blood flow is a physiological process through which an increase in vascular luminal pressure or stretch of the blood vessel wall causes vasoconstriction and sustained vascular tone [115]. Autoregulation is very dependent on the entry of extracellular Ca^2+^ via stretch-activated channels [116]. In comparison with VDCCs and ROCs, stretch-activated Ca^2+^ channels are more sensitive to the Ca^2+^ antagonist diltiazem but not to dihydropyridines. In SMC membranes, mechanical stretch stimulates ^45^Ca^2+^ influx [117]. Also, studies in cannulated cat cerebral artery with intact endothelium showed that elevation of intramural pressure was associated with membrane depolarization, action potential generation, and vasoconstriction. Interestingly, chemical disruption of ECs without damaging VSMCs by briefly perfusing the vessels with collagenase and elastase abolished the response to increased intramural pressure, suggesting a role of ECs as a transducer of autoregulation and the vascular response to pressure [118].

Stretch-activated Ca^2+^ channels include TRP vanilloid type 2 (TRPV2) [119]. Also, TRPV4 is activated by mechanical stimuli, shear stress, and expansion of cell volume and swelling and is an important mediator of myogenic tone [74]. TRPC6 is also a mechanosensitive channel that is activated by shear stress and cell swelling and promotes Ca^2+^ influx into VSMCs and vasoconstriction [120]. A mutant TRPC6 has been associated with familial focal segmental glomerulosclerosis [121]. Other mechanosensitive Ca^2+^ channels include TRPPs [84]. Also, Piezo-type channel is a superfamily of mechanosensitive cation channels responsible for stretch-activated Ca^2+^ and Na^+^ influx in multiple cell types [122].

## 5. Mechanisms of Ca^2+^ Removal

The vascular cell plasma membrane and intracellular organelles are multifunctional. In addition to the role of plasma membrane channels in Ca^2+^ entry, the vascular cell PMCA and Na^+^/Ca^2+^ exchanger contribute to removal of excess [Ca^2+^]_c_ to the extracellular space (Figure 1). Likewise, the ER and SR not only have Ca^2+^ release channels but also Ca^2+^ uptake mechanisms via SERCA. Also, the mitochondrial membranes regulate [Ca^2+^]_c_ through a pump-leak system involving rescue Ca^2+^ uptake of excess Ca^2+^ from the cytosol and passive, slow Ca^2+^ leak back to the cytosol under resting conditions.

### 5.1. Plasmalemmal Ca^2+^-ATPase (PMCA)

In smooth muscle of guinea pig taenia coli, treatment with metabolic inhibitors such as 2,4-dinitrophenol or iodoacetic acid causes a net accumulation of Ca^2+^ equivalent to the passive Ca^2+^ leak [123,124], suggesting that Ca^2+^ removal via an ATP-dependent pathway participates in SMC Ca^2+^ ion homeostasis and that suppression of this pathway causes cellular accumulation of Ca^2+^ [125]. The SMC PMCA shares some of the characteristics of the Ca^2+^ extrusion pump in red blood cells and the squid axon [126]. PMCA is a large enzyme (~130 kDa) that belongs to the P-type ATPase family, which also includes SERCA. PMCA ultrastructure comprises ten transmembrane domains and a C-terminus cytosolic tail that harbors the CaM-binding and regulatory regions [127]. PMCA is distinguished from other plasma membranes and ER/SR ATPases by being insensitive to ouabain (different from Na^+^/K^+^-ATPase), having reduced sensitivity to K^+^ (less sensitive than SERCA), and being sensitive to CaM inhibitors and vanadate inhibition (more sensitive than SERCA) [128]. PMCA plays a major role in regulating [Ca^2+^]_c_ and vascular tone. PMCA inhibition by vanadate causes maximal VSMC contraction [129]. Also, oxytocin and prostaglandins promote SMC contraction partly through PMCA inhibition [130,131]. PMCA has been cloned and purified, and its amino acid sequence has been identified in several cell types, including SMCs [132,133,134].

During the Ca^2+^ transport cycle, PMCA undergoes conformational changes that utilize ATP hydrolysis to pump cytosolic Ca^2+^ to the extracellular space against a steep concentration gradient. In its E1 state, the PMCA cytosolic side binds Ca^2+^ with high affinity, triggering ATP binding and autophosphorylation of a conserved aspartate residue in its phosphorylation P-domain [135]. PMCA phosphorylation induces a conformational change from the E1 to the E2 state with lower Ca^2+^ affinity, thus allowing Ca^2+^ release to the extracellular space. Following Ca^2+^ extrusion, PMCA undergoes dephosphorylation and returns to the E1 state to begin another transport cycle [136].

PMCA function is modulated by several factors, most notably the Ca^2+^-binding protein CaM. Upon binding to Ca^2+^, the Ca^2+^-CaM complex activates PMCA by interacting with a specific regulatory domain on the cytoplasmic side, enhancing PMCA’s affinity for Ca^2+^ and accelerating its rate of Ca^2+^ extrusion [137]. CaM-mediated activation is crucial following neuronal or muscle excitation and increases in [Ca^2+^]_c_ in order to rapidly extrude Ca^2+^ and restore baseline [Ca^2+^]_c_. Under resting conditions and in the absence of interaction with CaM, PMCA maintains a basal level of activity and provides a steady, low-efflux Ca^2+^ clearance mechanism [137,138].

PMCA has different isoforms and expression patterns in different tissues, allowing it to meet the distinct Ca^2+^ clearance needs of different cell types. PMCA-2 and PMCA-3 are highly expressed in excitable muscle cells and neurons, where rapid Ca^2+^ clearance is critical for proper function. In contrast, PMCA-4 is more ubiquitously expressed and provides slower and sustained Ca^2+^ extrusion in other tissues [139].

### 5.2. Sarco(endo)plasmic Reticulum Ca^2+^-ATPase (SERCA)

SERCA is another ATP-activated pump that actively transports Ca^2+^ from the cytoplasm into ER/SR to maintain low [Ca^2+^]_c_ under resting conditions, reduce [Ca^2+^]_c_ following cell activation, and sustain replenished Ca^2+^ stores in preparation for the next cell stimulation. SERCA’s role in maintaining Ca^2+^ homeostasis has been well-defined in the heart and skeletal muscle [140]. SERCA (100 kDa) has a 2:1 stoichiometry ratio, transporting two Ca^2+^ ions for hydrolysis of one ATP molecule. SERCA has an adequate affinity for Ca^2+^ (K_m_ = 0.2–0.6 μM), allowing it to accumulate Ca^2+^ and facilitate muscle relaxation. Like PMCA, SERCA functions in cycles of conformational changes to transport Ca^2+^ against its concentration gradient. In its unphosphorylated E1 state, SERCA faces the cytosolic side of the ER/SR membrane, where it binds two Ca^2+^ ions at high-affinity regions in its transmembrane domains. Ca^2+^ binding causes conformational changes that attract ATP to the SERCA nucleotide-binding N-domain, triggering autophosphorylation at a conserved aspartate residue in the phosphorylation P-domain [141]. SERCA phosphorylation causes its transition from the E1-P to the E2-P state with reduced Ca^2+^ affinity that facilitates translocation of Ca^2+^ ions from the cytosol to ER/SR lumen. Upon releasing Ca^2+^ ions into ER/SR, SERCA undergoes dephosphorylation and resetting to its E1 state to initiate a new cycle [142].

The SR ability to take-up Ca^2+^ is less in SMCs in comparison to cardiac and skeletal muscle [143]. Nevertheless, SMC SR microsomes exhibit ATP-dependent Ca^2+^ accumulation. Electron probe analysis of smooth muscle preparations has also revealed a non-mitochondrial Ca^2+^ uptake pathway that depends on ATP and can be inhibited using vanadate [32].

SERCA is regulated by multiple proteins that adjust its activity to meet cellular demands. Calsequestrin is a low-affinity but high-capacity Ca^2+^-binding protein that enhances the Ca^2+^ storing capacity of SR in SMCs and skeletal muscle [144,145]. Phospholamban (PLN) is a major regulatory protein in cardiac muscle. In its resting, un-phosphorylated form, PLN binding to SERCA reduces its affinity to Ca^2+^ and effectively inhibits the pump activity. During sympathetic activation and β-adrenergic stimulation, increases in adenylate cyclase, cAMP, and protein kinase A (PKA) activity cause PLN phosphorylation, relieving PLN inhibition of SERCA and enabling rapid Ca^2+^ reuptake into SR. This is critical for both cardiac muscle relaxation (diastole) and the preparation for the next contraction (systole), thus facilitating increases in heart rate during stress and exercise [146]. During cold exposure, sarcolipin, a small regulatory protein in skeletal muscle, binds to SERCA and induces “uncoupling” or ATP hydrolysis without Ca^2+^ translocation, thus generating heat instead of storing Ca^2+^ in SR, and contributing to non-shivering thermogenesis and maintaining body temperature under cold stress. Sarcolipin-mediated uncoupling also helps to buffer energy utilization in muscle cells by modulating ATP demand during periods of cellular stress [147]. Post-translational modifications, including N-glycosylation, glutathionylation and Ca^2+^/CaM-dependent protein kinase II (CaMKII)-mediated phosphorylation, affect SERCA pump activity [148]. Also, cyclopiazonic acid is a specific SERCA inhibitor.

### 5.3. Sodium/Calcium Exchanger (NCX)

The NCX is an important surface membrane mechanism that can help in removing excess [Ca^2+^]_c_ to the extracellular environment against a large transmembrane Ca^2+^ concentration gradient. NCX participates in removal of intracellular Ca^2+^ in numerous tissues and cells including SMCs [149,150]. Studies on isolated membrane vesicles were able to co-purify NCX activity with markers of the surface membrane, supporting its location at the plasma membrane. Other studies were able to isolate, functionally reconstitute [151,152], and distinguish plasmalemmal NCX from mitochondrial NCX based on differences in their stoichiometry and specificity [153].

NCX activity is propelled by the transmembrane concentration gradient of Na^+^ and Ca^2+^ and the surface membrane potential. The energy harnessed from the movement of Na^+^ or Ca^2+^ down its concentration gradient is counterbalanced by an anti-port transport of the coupled ion with a stoichiometry of 3Na^+^:Ca^2+^, resulting in an electrogenic pathway [154,155]. NCX participates in Ca^2+^ extrusion from VSMCs with a variable role in various vascular beds [156,157]. Importantly, NCX could function in a bidirectional fashion. Depending on the transmembrane Na^+^ and Ca^2+^ concentration gradient, and the surface membrane potential, NCX mainly functions as a Ca^2+^ removal pathway (forward-mode), but could function as a Ca^2+^ entry pathway (reverse-mode) with an increasing role in pathological elevation of [Ca^2+^]_c_ in cardiovascular disease and HTN [158].

### 5.4. Superficial Ca^2+^ Gradient (Buffer Barrier)

The Ca^2+^ uptake and extrusion mechanisms could function in an elaborate cooperative fashion, such that excess extracellular Ca^2+^ leak or depolarization-induced Ca^2+^ influx is taken first by the SR located close to the plasma membrane, then extruded largely toward the cell surface and outside the cell through PMCA or NCX, thus generating a peripheral subplasmalemmal Ca^2+^ gradient or “superficial buffer barrier”. This cooperative pathway has been postulated and studied extensively in VSMCs [159]. The superficial buffer barrier hypothesis has two predictions: (1) The proportion of Ca^2+^ crossing the plasmalemma and taken up by superficial SR is critical in defining the threshold Ca^2+^ influx that causes contraction. (2) There is an irregular outwardly-directed Ca^2+^ gradient in a narrow (~10 nanometer) region just underneath the plasma membrane. The first prediction was supported by the observation that depleting SR Ca^2+^ by stimulating aortic segments with norepinephrine in Ca^2+^-free medium diminished the ability of high KCl depolarization-mediated Ca^2+^ influx to induce contraction. High KCl-induced contraction was restored when the aortic rings were bathed in Ca^2+^-containing solution to replenish the SR Ca^2+^ store to its normal capacity. These observations suggested that the buffering of Ca^2+^ influx prevented Ca^2+^ from reaching the deeper myoplasm [160]. Also, the ^45^Ca^2+^ influx-contraction relationship was shifted to the left in aortic rings stimulated with norepinephrine compared to those stimulated by high KCl. In other words, in order to achieve the same contraction, more Ca^2+^-influx was required during high-KCl-induced depolarization than during norepinephrine-induced activation [161], supporting the idea that a proportion of KCl-induced Ca^2+^ influx is taken by the superficial SR before activating the myofilament. In contrast, most of the norepinephrine-induced Ca^2+^ influx is available for the myofilament because of the increased SR permeability and its inability to accumulate Ca^2+^. Also, SERCA inhibition by cyclopiazonic acid causes slowly developing contractions in VSM, with successive applications causing smaller repeatable contractions depending on the vessel type. In rat aorta, a second cyclopiazonic acid-induced contraction is decreased but is completely repeatable after treatment with the PMCA inhibitor vanadate or by inhibiting NCX forward-mode using 2′,4′-dichlorobenzamil but not with the Na^+^/K^+^ pump inhibitor ouabain or the reverse-mode NCX inhibitor KBR7943. These observations suggest that the cyclopiazonic acid-induced slow increase in [Ca^2+^]_c_ causes prolonged stimulation of Ca^2+^ removal by PMCA or NCX that diminishes its ability to induce repeated contractions, providing evidence for functional coupling between SERCA and plasmalemmal Ca^2+^ extrusion mechanisms in VSMCs [162].

The second prediction of the superficial buffer barrier hypothesis is supported by identifying an uneven dispersion of intracellular Ca^2+^ in subcellular and nanojunctional regions between the SR and surface membrane and other cellular organelles, including lysosomes, mitochondria, and the nucleus [163,164]. Also, in fura-2-labelled single VSMCs from myogenic cerebral arteries, Ca^2+^ sparks were detected as localized, spontaneous, and ryanodine-responsive increments in [Ca^2+^]_c_ from SR slightly underneath the plasma membrane. This superficial Ca^2+^ gradient of high fluorescence signal would activate Ca^2+^-dependent K^+^ channels and promote vascular relaxation [165].

### 5.5. Ca^2+^ Regulation by Mitochondria

The SR has a finite capacity to take up Ca^2+^, making the mitochondria an alternative Ca^2+^ reservoir during successive and extraordinary Ca^2+^ burdens [166]. Mitochondria represent ~5% of SMC volume and use both Ca^2+^ uptake and Ca^2+^ efflux pathways to regulate the cell response to metabolic demands and stresses [153,167]. Ca^2+^ uptake/influx occurs through the mitochondrial Ca^2+^ uniporter (MCU) complex, which comprises a pore-forming subunit/channel and associated regulatory proteins. MCU is highly selective for Ca^2+^ and is influenced by both the electrochemical gradient across the inner mitochondrial membrane and the large inside −150 mV membrane potential of mitochondria. Ca^2+^ efflux has a lower capacity than Ca^2+^ influx [167] and is facilitated by electrogenic Ca^2+^:3Na^+^ [167] and electroneutral Ca^2+^:2H^+^ antiporter [168,169]. Along with Ca^2+^, mitochondria also take up phosphate anions through an HPO_4_^2−^:2OH^−^ exchange, leading to the formation of calcium phosphate. Mitchell’s paradigm of mitochondria energy transfer [170] predicts an initial process involving the formation of a proton electrochemical gradient across the mitochondrial membrane, leading to a greater alkaline pH in mitochondria versus the cytosol that lowers the solubility of calcium phosphate. Consequently, the level of free Ca^2+^ in the mitochondrial matrix is determined by the amount of extra- and intra-mitochondrial phosphate, the intra-mitochondrial pH, and the K_m_ and V_max_ of the Ca^2+^ efflux pathways [168]. The rate of mitochondrial Ca^2+^ influx markedly accelerates as [Ca^2+^]_c_ increases to dangerously extreme levels, allowing the mitochondria to function as a sink for Ca^2+^ overload. Since mitochondrial Ca^2+^ efflux is slow and saturable [167], the rate of Ca^2+^ influx will surpass Ca^2+^ efflux, leading to accumulation of Ca^2+^ and formation of intra-mitochondrial calcium phosphate [168]. However, the mitochondrial free Ca^2+^ concentration is in equilibrium with the sizable nonionic calcium phosphate reservoir and [Ca^2+^]_c_ such that when [Ca^2+^]_c_ is less than mitochondrial free Ca^2+^ concentration, the nonionic calcium reservoir is mobilized to maintain resting [Ca^2+^]_c_. Mitochondrial efflux of Ca^2+^ and other ions occurs mainly through the mitochondrial permeability transition pore (mPTP). Importantly, the opening of the mPTP and excessive mitochondrial Ca^2+^ release could cause mitochondrial swelling, loss of membrane integrity, cellular stress, oxidative damage, high cellular Ca^2+^ concentration, apoptosis, and death [171]. Other regulatory proteins such as MICU1–3 heterodimers function as gatekeepers for the MCU complex to ensure that mitochondrial Ca^2+^ uptake occurs only at abnormally high [Ca^2+^]_c_. At resting [Ca^2+^]_c_, MICU1–3 subunits block the pore and keep MCU low, but at dangerously high [Ca^2+^]_c_, they undergo conformational rearrangements and facilitate Ca^2+^ access to the pore and rapid Ca^2+^ uptake into the mitochondrial matrix. MICU1–3 dysfunction leads to aberrant mitochondrial Ca^2+^ uptake and has been linked to neurodegenerative disorders [172,173]. The MCU complex also interacts with mitochondrial Na^+^- and Li^+^-dependent Ca^2+^ exchangers, thus providing a feed-back loop that helps to maintain mitochondrial Ca^2+^ homeostasis and the balance between Ca^2+^ uptake and efflux, particularly during high metabolic activity and stress [173]. In contrast, when [Ca^2+^]_c_ is at its normal resting level (~0.1 μmol/L), and the free mitochondrial Ca^2+^ has an equal level, PMCA and SERCA will be in charge of maintaining the cellular Ca^2+^ homeostasis. Also, the large mitochondrial Ca^2+^ capacity is still finite, allowing the mitochondria to gradually release their stored calcium load during cell quiescence so that it can be removed by PMCA and SERCA. The apparent K_m_ of mitochondrial Ca^2+^ uptake (~10–17 μmol/L) is greater than SR K_m_ (~1 μmol/L), making SR the primary Ca^2+^ storing site under physiological circumstances, while the mitochondria function as an alternative Ca^2+^ sink only when [Ca^2+^]_c_ is abnormally high, exceeding 5 μmol/L [32,174]. Like most vascular cells, SMC mitochondria minimally accumulate Ca^2+^ under physiological circumstances, and the mitochondria’s great ability to buffer Ca^2+^ becomes critical under pathological circumstances during Ca^2+^ burdens and when excessive influx of extracellular Ca^2+^ threatens cell viability. In support, the isolated mitochondrial fraction from atherosclerotic vascular beds shows a high Ca^2+^ content, reflecting the initial sites of damaged SMCs and vascular calcification [32].

## 6. Ca^2+^ Regulation of Cell Growth and Proliferation

Ca^2+^ is a critical second messenger that regulates numerous cellular functions, including growth and proliferation. Precise control of [Ca^2+^]_c_ is essential for activating the signaling pathways that drive cell cycle progression, gene transcription, and metabolic processes. Ca^2+^ signaling occurs through oscillatory waves, localized microdomains, and sustained cytosolic increases, each providing spatially and temporally distinct signals that support the complex demands of cell growth and division [175,176]. Dysregulated Ca^2+^ signaling is implicated in cancer and other diseases where aberrant Ca^2+^ dynamics lead to uncontrolled cell growth and proliferation [177].

Ca^2+^ signaling for cell growth and proliferation is initiated through ligand binding to cell surface receptors, such as growth factor receptors, GPCRs, and receptor tyrosine kinases. The ligand–receptor interactions activate Ca^2+^ release from ER/SR and Ca^2+^ influx through plasma membrane channels, which increase [Ca^2+^]_c_ and downstream signaling. In the cytosol, Ca^2+^ binds to CaM, which activates several kinases and phosphatases, including CaM-dependent protein kinase and calcineurin. Protein kinases can then phosphorylate downstream targets involved in gene expression and cell cycle progression, such as extracellular signal-regulated kinases (ERK). On the other hand, phosphorylation of the transcription factor NFAT keeps it inactive in the cytosol, while calcineurin functioning as a Ca^2+^/CaM-regulated phosphatase can dephosphorylate and activate NFAT and thereby induce its translocation to the nucleus, where it promotes target gene expression [178,179]. Also, CaMKII regulates transcription factors like cAMP response element-binding protein (CREB), promoting the expression of genes necessary for cell proliferation [180,181]. Excessive [Ca^2+^]_c_ also stimulates mitochondrial uptake via MCU, leading to the increased production of ATP to support the energy demands for cell growth. Also, Ca^2+^-regulated mitochondrial production of reactive oxygen species (ROS) serves as a second messenger to promote signaling pathways essential for cell proliferation [182].

**Figure 2:**
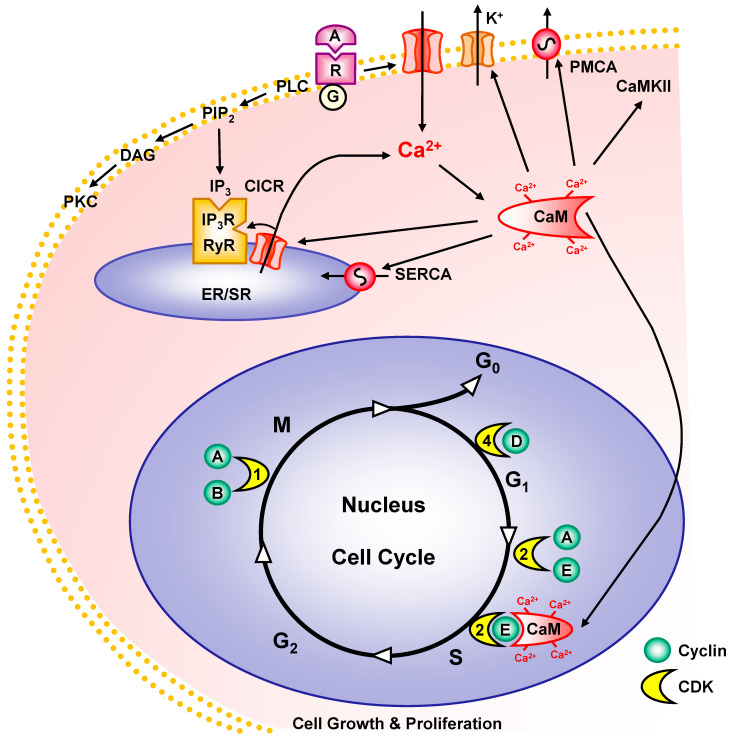
Ca^2+^-dependent vascular cell proliferation. In vascular cells, a mitogenic agonist such as vascular endothelial growth factor (VEGF) or Ang II (A) interacts with its receptor (R) and increases the breakdown of phosphatidylinositol 4,5-bisphosphate (PIP_2_) and the generation of IP_3_. IP_3_ triggers Ca^2+^ release from ER/SR. Agonists also stimulate Ca^2+^ entry from the extracellular space via Ca^2+^ channels. Ca^2+^ binding to calmodulin (CaM) activates myosin light chain kinase (MLCK) and stimulates VSMC contraction or controls the activity of K^+^ channels, SR Ca^2+^ release, SERCA, PMCA, and CaM kinase II. In response to vascular injury, differentiated vascular cells transform into synthetic phenotype and enter a cell cycle comprising different stages: G_1_ (growth and alignment of chromosomes for replication), S (DNA synthesis), G_2_ (preparation for imminent mitosis), and M (mitosis). Ca^2+^/CaM promotes cyclin E/cyclin-dependent kinase 2 (CDK2) activity, stimulates G_1_/S transition, and consequently enhances vascular cell proliferation.

The cell cycle is regulated by cytosolic proteins termed cyclins, which play a role in important processes such as wound repair and the response of vascular and cardiac tissues to injury and inflammation [183] (Figure 2). Cyclins target and activate cyclin-dependent kinases (CDKs), which in turn phosphorylate various proteins that control the cell cycle. The cellular CDK levels are relatively stable, but the levels of cyclins rise and fall depending on the cell cycle stage. Cyclin D and CDK4 primarily regulate the G_1_ phase, and cyclins A and E and CDK2 regulate the S-phase, whereas cyclins A and B and CDK1 regulate mitosis. Growth factors upregulate and enhance tyrosine phosphorylation of ERK-1, which promotes cyclin expression and VSMC growth [184]. A decrease in ERK1/2 phosphorylation reduces cyclin D1 and E expression, leading to the arrest of the cell cycle at the G_1_ phase and reduced VSMC proliferation [185].

Ca^2+^ is critically involved in all stages of the cell cycle, from G1 to mitosis. In the G1 phase, Ca^2+^ influx and [Ca^2+^]_c_ oscillations activate cyclin D and CDKs, driving progression toward the G1/S transition. [Ca^2+^]_c_ oscillations further influence cell cycle checkpoints by modulating retinoblastoma protein and the transcription factor E2F, both of which are necessary for S-phase entry [186,187]. Elevated [Ca^2+^]_c_ is essential for mitotic spindle formation and chromosome alignment. Mitotic exit is also Ca^2+^-dependent, as Ca^2+^ influx promotes the dephosphorylation of mitotic kinases, enabling cytokinesis and cell division [188].

Given the importance of Ca^2+^ in cell growth and proliferation, dysregulated Ca^2+^ signaling is a hallmark of cancer. Many cancer cells exhibit altered Ca^2+^ homeostasis, which enables sustained proliferation and resistance to cell death signals. Overexpression of Ca^2+^ channels, such as TRP and Orai1 channels, has been observed in various cancers, contributing to persistent Ca^2+^ signaling that drives oncogenic pathways [189,190]. Increased VSMC proliferation is also a major factor in intimal hyperplasia, peripheral artery disease and vascular restenosis following angioplasty, and targeting Ca^2+^ signaling pathways, either by Ca^2+^ channel blockers or modulators of the activity of Ca^2+^-dependent kinases, could be useful in mitigating these conditions [191,192].

## 7. Ca^2+^ Regulation of Endothelial Cell Function

The endothelium regulates vascular tone, barrier integrity, and inflammatory responses, and Ca^2+^ signaling is indispensable to these roles. Agonist interaction with its GPCR causes increases in [Ca^2+^]_c_ due to Ca^2+^ mobilization from ER and Ca^2+^ entry via various Ca^2+^ channels, including store-operated Ca^2+^ entry. Also, TRPV4 and TRPC4 respond to shear stress from blood flow, allowing Ca^2+^ entry into the cell and triggering downstream pathways that promote EC alignment to blood flow changes and contribute to vasodilation.

### 7.1. Ca^2+^ Regulation of Endothelial Nitric Oxide (NO) Production

**Figure 3:**
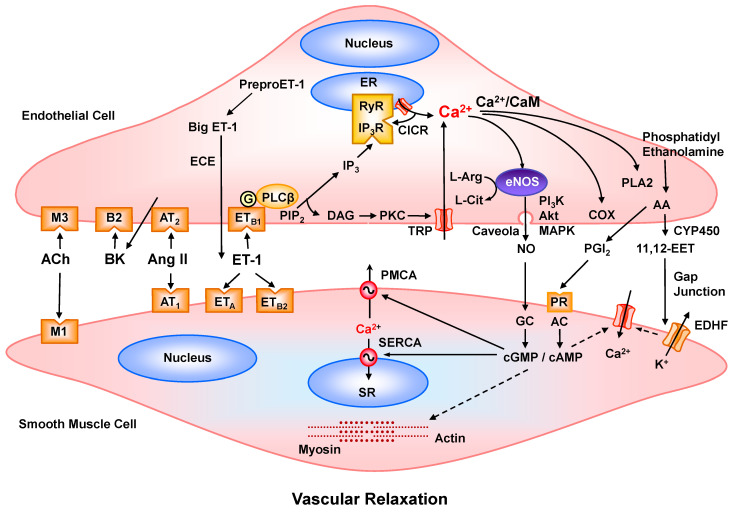
Ca^2+^ regulation of endothelium-dependent vascular relaxation. Vasodilators such as acetylcholine (ACh) via muscarinic receptor (M3), bradykinin (BK) via B2 receptor, Ang II via AT_2_R or through releasing BK, and endothelin-1 (ET-1) via endothelin B type 1 receptor (ET_B1_R) activate GTP-binding protein (G) and phospholipase C (PLCβ) to increase the hydrolysis of phosphatidylinositol 4,5-bisphosphate (PIP_2_) into inositol 1,4,5-triphosphate (IP_3_) and diacylglycerol (DAG). IP_3_ stimulates Ca^2+^ release from the endoplasmic reticulum (ER), and DAG stimulates protein kinase C (PKC) and enhances Ca^2+^ influx via Ca^2+^ channels. Increased [Ca^2+^]_c_ increases the activity of eNOS, which promotes the transformation of L-arginine (L-Arg) to L-citrulline (L-Cit) and the generation of NO. NO enters VSMCs and stimulates guanylate cyclase to produce cyclic guanosine monophosphate (cGMP), which reduces [Ca^2+^]_c_ by inhibition of Ca^2+^ influx via Ca^2+^ channels and stimulation of sarco(endo)plasmic reticulum Ca^2+^-ATPase and uptake pump (SERCA) and the plasma membrane Ca^2+^-ATPase and extrusion pump (PMCA) and decreases the force sensitivity of the actin–myosin myofilaments to [Ca^2+^]_c_, resulting in VSMC relaxation. Vasodilator-induced increases in EC [Ca^2+^]_c_ also promote the activity of cyclooxygenases (COX) and increase the production of prostacyclin (PGI_2_), which activates the VSMC prostanoid receptor (PR) and stimulates adenylate cyclase to increase the formation of cyclic adenosine monophosphate (cAMP), which activates pathways similar to those activated by cGMP to decrease [Ca^2+^]_c_ and the force sensitivity of the contractile myofilaments to Ca^2+^, resulting in further VSMC relaxation. Vasodilators can also enhance endothelium-derived hyperpolarizing factor (EDHF) by activating the myo-endothelial gap junctions or promoting the release of epoxyeicosatrienoic acids (EETs), hydrogen peroxide (H_2_O_2_), K^+^, or C-type natriuretic peptide (CNP), resulting in activation of VSMC K^+^ channels, plasma membrane hyperpolarization, reduced Ca^2+^ influx via Ca^2+^ channels and VSMC relaxation.

One of the critical roles of Ca^2+^ in ECs is activation of endothelial NO synthase (eNOS), an enzyme responsible for NO production. In resting conditions, eNOS is bound to caveolin in the surface membrane caveolae. Stimulation of ECs by vasodilators such as acetylcholine (ACh) or bradykinin causes increases in [Ca^2+^]_c_ (Figure 3). Ca^2+^ binds to CaM, a Ca^2+^-sensitive protein that initiates eNOS dissociation from caveolin. ACh or bradykinin also activate mitogen-activated protein kinase (MAPK) and phosphatidylinositol-3-kinase (PI_3_K)/Akt, which promote the phosphorylation and redistribution of cytoplasmic eNOS back to the surface membrane, where it is myristoylated, palmitoylated, and fully activated [193,194,195]. Activated eNOS, in the presence of cofactors such as tetrahydrobiopterin (BH4) and nicotinamide adenine dinucleotide phosphate (reduced form) (NADPH), stimulates the conversion of L-arginine to L-citrulline and generation of NO [196]. NO enters into adjacent VSMCs, where it activates soluble guanylyl cyclase (sGC) and enhances the formation of cyclic guanosine monophosphate (cGMP) [196]. In VSMCs, cGMP stimulates protein kinase G (PKG), which activates K^+^ channels, causing membrane hyperpolarization and inhibition of VDCCs and in turn limiting Ca^2+^ influx and promoting relaxation. PKG also stimulates Ca^2+^ uptake by SERCA and Ca^2+^ extrusion by PMCA and phosphorylates and inhibits myosin light chain kinase (MLCK), thus reducing MLC phosphorylation and promoting further relaxation.

Ca^2+^-mediated NO production has broader implications for cardiovascular health. NO not only promotes vasodilation but also prevents platelet aggregation and leukocyte adhesion to ECs and thereby protects against vascular thrombosis and atherosclerosis [197]. Reduced NO bioavailability, often resulting from impaired Ca^2+^ signaling, is linked to endothelial dysfunction, a common feature in HTN, diabetes, and atherosclerosis.

### 7.2. Ca^2+^ Regulation of Endothelial Prostacyclin Release

EC Ca^2+^ signaling plays a role in the synthesis of prostacyclin (PGI_2_), which promotes vasodilation, inhibits platelet aggregation, and thereby maintains vascular homeostasis and reduces the risk of thrombosis. PGI_2_ synthesis involves phospholipase A2 (PLA2) and cyclooxygenase-1 (COX-1), both of which require Ca^2+^ for activation and subsequent generation of arachidonic acid and PGI_2_.

Agonist- and shear stress-induced increases in [Ca^2+^]_c_ activate PLA2, which stimulates the breakdown of the membrane phospholipid phosphatidylethanolamine and the generation of arachidonic acid. PKC-mediated phosphorylation enhances PLA2 activity and substrate binding [198,199]. EC [Ca^2+^]_c_ facilitates arachidonic acid metabolism and the generation of PGI_2_ through the activation of COX and PGI_2_ synthase [200]. Ca^2+^ enhances the expression/activity of COX-1, which catalyzes the transformation of arachidonic acid into an unstable intermediate termed prostaglandin H_2_ (PGH_2_), a reaction that requires molecular oxygen and involves peroxidase activity within the COX enzyme. PGH_2_ is subsequently converted by EC PGI_2_ synthase into PGI_2_, which is released into the circulation, where it exerts vasodilatory and antithrombotic effects. PGI₂ activates prostanoid receptors in VSMCs leading to the activation of adenylate cyclase and generation of cAMP, which activates PKA and promotes VSMC relaxation pathways similar to those activated by cGMP and PKG. PGI_2_ also targets platelets, causing the inhibition of platelet aggregation and reducing thrombosis [201].

### 7.3. Ca^2+^ Regulation of Endothelin-1 (ET-1) Synthesis

ET-1 is one of the most potent vasoconstrictors. ET-1 is produced by ECs and participates in the regulation of vascular tone, cell proliferation, and inflammation. Ca^2+^ stimulates mRNA transcription and the secretion of ET-1 in response to various physiological and pathological stimuli. Increases in EC [Ca^2+^]_c_ stimulate NFAT and activator protein-1 (AP-1), two transcription factors involved in *EDN1* gene activation. NFAT redistributes to the nucleus, where it binds to the ET-1 gene promoter regions and enhances its transcription [202]. Following transcription, ET-1 is synthesized as a precursor prepro-ET-1, which is cleaved in ER by a signal peptidase into pro-ET-1, then by furin-like enzyme into big ET-1 [203]. Endothelin-converting enzyme (ECE), a metalloprotease located in the plasma membrane, then converts big ET-1 into mature ET-1, which is secreted from ECs to act on nearby SMCs and other target tissues. [Ca^2+^]_c_ regulates ET-1 production by influencing ET-1 precursor availability and secretion, as well as regulating the activity of ECE and Ca^2+^-dependent metalloproteases [204,205].

ET-1 exerts its effects by binding to ET_A_R and ET_B_R. ET_A_R is predominantly expressed in VSMCs and mediates vasoconstriction and increases in BP, while ET_B1_R in ECs promotes NO release and vasodilation. Under pathological conditions, ET_B2_R is also expressed in VSMCs and contributes to vasoconstriction [203]. ET-1 also activates MAPK and Rho-kinase (ROCK) signaling pathways, thus promoting VSMC proliferation and inflammatory responses. Ca^2+^-dependent activation of these pathways further contributes to vascular remodeling and fibrosis, as observed in atherosclerosis and heart failure [206,207]. Endothelial dysfunction, a hallmark of cardiovascular disease, often involves imbalance between NO production and ET-1 release. Reduced NO bioavailability combined with excessive ET-1 release promote a pro-constrictive and pro-inflammatory state that exacerbates vascular damage [208].

### 7.4. Ca^2+^ Regulation of Mitochondrial Enzymes and Oxidative Stress

Ca^2+^ plays a role in regulating mitochondrial enzymes, thus aligning energy production with cellular demand. Mitochondrial Ca^2+^ uptake activates key enzymes in the tricarboxylic acid (TCA) cycle, including pyruvate dehydrogenase, isocitrate dehydrogenase, and α-ketoglutarate dehydrogenase, which accelerate ATP production and support energy demand for different processes such as cell division and repair [173]. Mitochondrial Ca^2+^ regulation also influences the generation of ROS, impacting oxidative stress and signaling pathways critical for cell survival and function [209,210]. ROS include superoxide (O_2_^•−^) and hydrogen peroxide (H_2_O_2_). Membrane-bound NADPH oxidase catalyzes the one-electron transfer and O_2_ reduction to O_2_^•−^. NOX1 is a NADPH oxidase isoform that shows overexpression in some forms of HTN [211]. Excess O_2_^•−^ formation would scavenge EC NO to produce peroxynitrite (ONOO^−^), resulting in reduced NO bioavailability, oxidative stress and EC damage [212].

### 7.5. Ca^2+^ Regulation of Vascular Permeability

The EC barrier is a critical regulator of vascular permeability, which allows selective passage of substances across the vascular wall. Ca^2+^ plays an essential role in controlling EC barrier integrity [197]. Ca^2+^ influences adherens junctions and tight junctions between ECs. Increases in [Ca^2+^]_c_ disrupts the stability of vascular endothelial (VE)-cadherin, promoting gap formation and increasing permeability [213]. During inflammation, Ca^2+^-mediated EC barrier disruption facilitates the transmigration of immune cells and soluble mediators into the surrounding tissue, promoting an inflammatory response [214]. While this process is necessary for immune responses, sustained Ca^2+^ elevation exacerbates EC barrier dysfunction, contributing to chronic inflammation and vascular leakage, as observed in acute respiratory distress syndrome and sepsis [215,216].

## 8. Ca^2+^ Regulation of VSMC Function

VSMC contraction and relaxation are central processes in the regulation of vascular tone, circulatory homeostasis and BP. Precise control of Ca^2+^ handling mechanisms including Ca^2+^ release, influx, storage, and removal, alongside the regulation of downstream enzymes and signaling pathways, determine the extent of contraction or relaxation of blood vessels in response to physiological demands and during pathological conditions.

### 8.1. Ca^2+^-Dependent Myosin Light Chain Phosphorylation

**Figure 4:**
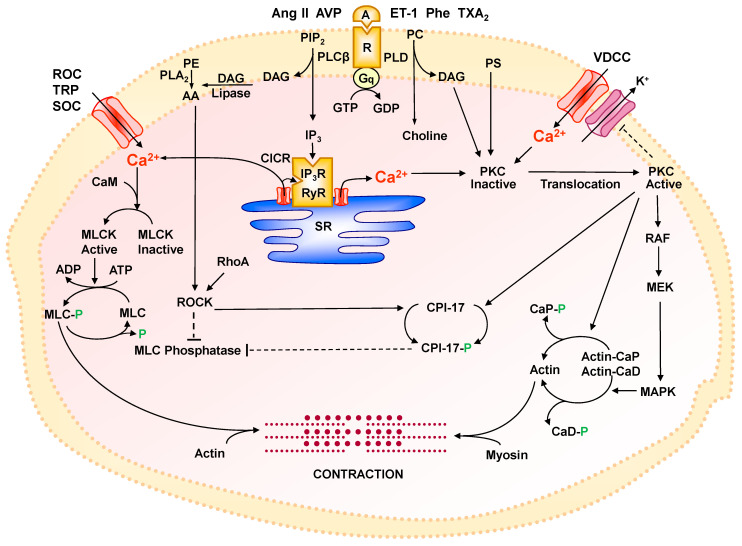
Ca^2+^ regulation of VSMC contraction. The interaction of a vasoconstrictor agonist (A) with its receptor (R) is coupled to guanosine triphosphate (GTP)-binding protein (Gq) and activation phospholipase C (PLCβ), which stimulates the hydrolysis of phosphatidylinositol 4,5-bisphosphate (PIP_2_) into inositol 1,4,5-trisphosphate (IP_3_) and diacylglycerol (DAG). The agonist also activates phospholipase D (PLD), which hydrolyzes phosphatidylcholine (PC) into choline and DAG. IP_3_ stimulates Ca^2+^ release from the sarcoplasmic reticulum (SR). Vasoconstrictors also enhance Ca^2+^ influx via various types of Ca^2+^ channels. Ca^2+^ binding to calmodulin (CaM) and formation of Ca^2+^/CaM complex activate MLCK, stimulate MLC phosphorylation, and promote VSMC contraction. DAG together with phosphatidylserine (PS) and Ca^2+^ activate and translocate conventional protein kinase Cs (cPKCs) from the cytosol to the surface membrane. PKC can inhibit K^+^ channels, resulting in surface membrane depolarization and stimulation of voltage-dependent Ca^2+^ channels (VDCCs). PKC may also phosphorylate PKC-potentiated phosphatase inhibitor protein-17 (CPI-17), which was found to inhibit MLC phosphatase and enhance the force sensitivity of the contractile myofilaments to Ca^2+^. PKC can also phosphorylate and untether calponin (CaP), thereby facilitating actin binding to myosin. PKC activation may also trigger a cascade of factors and protein kinases, comprising Raf, MAPK kinase (MEK), and MAPK (ERK_1/2_), that ultimately lead to phosphorylation and untethering of the actin-binding protein caldesmon (CaD), further promoting actin–myosin interaction. Vasoconstrictors can also activate RhoA/ROCK, which inhibits MLC phosphatase and thereby enhances the myofilament force sensitivity to Ca^2+^. Increased formation of arachidonic acid (AA), due to DAG transformation by DAG lipase or Ca^2+^-induced activation of phospholipase A2 (PLA2) and hydrolysis of phosphatidylethanolamine (PE), can also activate Rho-kinase (ROCK), and further promote VSMC contraction. Dashed line indicates inhibition.

VSMCs are a major component of the tunica media of blood vessels, and VSMC contraction plays an important role in the regulation of peripheral vascular resistance and BP. Vasoconstrictor agonists such as norepinephrine, Ang II, and ET-1 activate their specific receptor and cause an initial contractile response that requires ATP-generated energy followed by a sustained contraction in the absence of substantial energy expenditure. Ca^2+^-dependent activation and phosphorylation of myosin light chain (MLC) and consequent actin–myosin cross-bridge cycling are major determinants of VSMC contraction [217,218]. VSMC contractile response is stimulated by an elevation of [Ca^2+^]_c_ due to initial Ca^2+^ mobilization from SR, followed by Ca^2+^ entry from the extracellular environment via plasmalemmal Ca^2+^ channels [18,28]. Ca^2+^ levels are multiple-fold greater in SR and the extracellular environment than in the cytoplasm, and when the Ca^2+^ channels in SR and plasma membrane open, they allow Ca^2+^ movement into the cytoplasm and increase [Ca^2+^]_c_. Ca^2+^ binding to CaM and the formation of Ca^2+^/CaM complex activate MLCK, which in turn phosphorylates the 20-kDa MLC [217,218]. Phosphorylated MLC then enhances the activity of actin-activated Mg^2+^-ATPase, resulting in actin–myosin interaction and VSMC contraction (Figure 4). ATP hydrolysis provides energy for cross-bridge cycling, generating the mechanical force required for VSMC contraction. VSMC relaxation occurs when [Ca^2+^]_c_ is decreased due to Ca^2+^ re-uptake by SERCA and Ca^2+^ removal via PMCA or NCX. The decrease in [Ca^2+^]_c_ allows the Ca^2+^/CaM complex to dissociate, and shifts the enzyme activity from MLCK and in favor of MLC phosphatase, which dephosphorylates the phosphorylated MLC, thus preventing further actin–myosin cross-bridge formation, terminating contraction, and allowing vascular relaxation [18].

### 8.2. Evidence for Other Mechanisms of VSMC Contraction

Ca^2+^-induced phosphorylation of MLC is not the only pathway that mediates agonist-stimulated vascular tone. In rabbit aorta segments bathed in Ca^2+^-free medium, α-adrenergic receptor agonists such as phenylephrine elicit an initial transient contractile response due to Ca^2+^ mobilization from SR followed by a markedly smaller but sustained contraction [28], which, in the absence of Ca^2+^ in the extracellular environment, suggests activation of Ca^2+^ sensitization pathways. Simultaneous recordings of contraction and [Ca^2+^]_c_ in VSMC tissues have supported agonist-induced increases in the Ca^2+^ sensitivity of the contractile myofilaments [8,219]. In isolated segments of rabbit inferior vena cava loaded with the Ca^2+^ indicator fura-2, α-adrenergic receptor activation by norepinephrine causes an initial contractile response, followed by a sustained contraction, and concomitantly produces a rapid peak in [Ca^2+^]_c_, followed by a smaller elevation in [Ca^2+^]_c_. By contrast, high KCl depolarizing solution causes large and maintained increases in both the contractile response and [Ca^2+^]_c_. Importantly, for the same increment in [Ca^2+^]_c_, norepinephrine causes a greater contractile response than that induced by high KCl. Also, the norepinephrine [Ca^2+^]_c_ force curve is augmented and shifted leftward of the high KCl-induced curve, providing evidence that norepinephrine increases the Ca^2+^ sensitivity of the contractile proteins [219].

Dissociations were also observed in the relationships between [Ca^2+^]_c_, MLC phosphorylation and force, making it difficult to explain all modalities of VSMC contraction by Ca^2+^-induced MLC phosphorylation. The previous belief of uniform cytosolic Ca^2+^ has been disputed by the finding of uneven intracellular Ca^2+^ levels in various subcellular regions [163,164]. Also, Ca^2+^ sensitization of the contractile myofilaments has been proposed as an alternative mechanism by which VSMC contraction is sustained without a corresponding increase in [Ca^2+^]_c_. This mechanism plays a crucial role in maintaining vascular tone under prolonged vasoconstrictive conditions and involves regulatory enzymes such as protein kinase-C (PKC) and Rho-kinase (ROCK).

### 8.3. Ca^2+^ Regulation of Protein Kinase-C (PKC)

Vasoconstrictors such as phenylephrine, Ang II, and ET-1 bind to specific GPCRs and activate a guanosine triphosphate (GTP)-binding protein and PLCβ, leading to the hydrolysis of PIP_2_ into IP_3_ and DAG [220]. IP_3_ promotes Ca^2+^ mobilization from SR, whereas DAG enhances PKC activity. PKC is a ubiquitous ser/thr kinase that phosphorylates multiple substrates in various cell types, including ECs, VSMCs, and fibroblasts [221,222]. Structurally, PKC is composed of an N-terminus regulatory domain, a hinge region, and a C-terminus catalytic domain [223]. On the basis of the molecular structure of the N-terminus domain, PKCs are categorized into conventional cPKCs (α, βI, βII, γ), novel nPKCs (δ, ε, η, θ), and atypical aPKCs (ζ, ι/λ) [20,47]. The cPKCs have four conserved regions (C1–C4) and five variable regions (V1–V5) and can be activated by Ca^2+^, DAG, and phosphatidylserine (PS). In nPKCs, a variant C2 region lacking the critical Ca^2+^-coordinating aspartic acid residues makes them insensitive to Ca^2+^ [224]. In comparison, aPKCs lack the C2 region but have a variant C1 region and therefore can be activated by PS but not by Ca^2+^ or DAG [224]. PKCμ and PKCν are viewed as a fourth class of PKC or as members of the protein kinase D (PKD) family [225,226]. In resting cells, PKC is mainly located in the cytosol, but upon binding to Ca^2+^ or DAG, it undergoes a conformational change and unfolding, thus increasing its hydrophobicity and association with plasma membrane lipids [227]. The cell membrane contains several focal adhesion domains alternating with caveolae. PKCα has been localized in caveolae [228], and annexins are PKC substrates that promote its plasma membrane association [229,230].

Nascent PKC is also phosphorylated by PKC kinase, phosphoinositide-dependent kinase (PDK), and mammalian target of rapamycin (mTOR) and could also be auto-phosphorylated in the activation loop, turn motif, and hydrophobic motif of the C-terminus domain [227,231,232]. PKC phosphorylation alters its protein conformation, electric charge, lipid affinity, and cell membrane binding and maintains it in a catalytically-active form. However, the open conformation of fully activated PKC could also increase its susceptibility to phosphatases and proteases, repetitive cycles of auto-phosphorylation/dephosphorylation, and proteolytic degradation and de novo synthesis [232,233].

Myristoylated, alanine-rich C kinase substrate (MARCKS) is a major PKC substrate that binds cytoskeletal F-actin and makes cross-bridges to the surface membrane [234]. PKC-mediated MARCKS phosphorylation generates an electrostatic charge that reduces its binding affinity and displaces it from the surface membrane. Other PKC substrates are involved in VSMC growth and contraction. Activation of PKC enhances the [Ca^2+^]_c_ sensitivity of the contractile proteins, thus maintaining VSMC contraction with little elevations in [Ca^2+^]_c_, reducing the need for constant Ca^2+^ influx, conserving energy expenditure, and enabling blood vessels to respond to prolonged vasoconstriction, sympathetic nervous system activation, and hormonal stimuli [21,82]. Of note, MLC phosphatase is comprised of a catalytic subunit and regulatory subunits such as myosin phosphatase target subunit 1 (MYPT1). PKC induces phosphorylation of PKC-potentiated phosphatase inhibitor protein-17 (CPI-17), which in turn binds to and inhibits the catalytic subunit of MLC phosphatase, thus increasing MLC phosphorylation and enhancing VSMC contraction [235]. PKC can also inhibit MLC phosphatase by phosphorylating MYPT1 [236]. Agonist-induced PKC activation may also lead to redistribution of the actin-binding protein calponin (CaP), thus reversing its inhibition of actin-activated myosin ATPase and enhancing VSMC contraction [237]. In support, CaP knockdown in ferret arterial VSM inhibits PKC-dependent contraction [19,238].

The activation of PKC may initiate a cascade of MAPK and other protein kinases that eventually stimulate VSMC growth or contraction [239,240]. In cultured VSMCs, mitogens cause the activation and translocation of cytosolic MAPK to the nucleus, where it promotes gene transcription and cell growth [241]. In differentiated contractile VSMCs, activated PKCε causes the translocation of cytosolic MAPK kinase (MEK) and MAPK to the surface membrane as a kinase complex. PKC then phosphorylates and activates MEK, which in turn phosphorylates and targets MAPK to the cytoskeleton, where it phosphorylates the actin-binding protein caldesmon (CaD), thus reversing its inhibition of Mg^2+^-ATPase activity and enhancing actin–myosin interaction and VSMC contraction [19,242]. Also, phenylephrine-induced activation of aortic VSM increases CaP-dependent auto-phosphorylation and activation of PKC and subsequently enhances MAPK activity, CaD phosphorylation, and VSMC contraction [243]. These PKC-mediated pathways are activated simultaneously with the increases in [Ca^2+^]_c_ and MLC phosphorylation to promote VSMC contraction [243]. Importantly, PKC activation by phorbol 12,13-dibutyrate (PDBu) does not directly alter the phospho-content of CaD [236], suggesting that they require other kinases downstream of PKC for their phosphorylation [243]. In esophageal smooth muscle, PKC-mediated contraction occurs in association with heat shock protein (HSP27)-linked p38 MAPK phosphorylation [244], lending support to a role of MAPK as a linking kinase in the signal transduction cascade between activated PKC in the plasma membrane and SMC contractile proteins.

PKC can also affect Ca^2+^ signaling dynamics through reciprocal feed-forward and feed-back mechanisms. In VSMCs, α1 adrenergic receptor stimulation induces Ca^2+^ release and simultaneously activates TRPV4-mediated Ca^2+^ influx and cell contraction through a PKC-dependent pathway [245]. PKC can also affect VSMC contraction by modulating the activity of plasmalemmal K^+^ channels and in turn the membrane potential and Ca^2+^ channel activity. Norepinephrine, Ang II, ET-1, serotonin, and neuropeptide Y activate PKC-mediated pathways leading to inhibition of K^+^ channels, membrane depolarization, and VSMC contraction [246,247,248]. In pulmonary arteries, PKC inhibition attenuates ET-1-induced constriction, [Ca^2+^]_c_, and store-operated Ca^2+^ entry, suggesting PKC contribution to not only Ca^2+^ sensitization but also Ca^2+^ influx [249]. PDBu-induced PKC activation also inhibits BK_Ca_ and stimulates vascular contraction [250,251,252,253] in coronary [254], pulmonary [251], cerebral [255], and uterine vessels [256]. PKC activation inhibits BK_Ca_ by inducing channel phosphorylation and decreasing its responsiveness to activation by PKG [257,258]. In rat mesenteric artery VSMCs, vasopressin modulates voltage-dependent K^+^ channel K_v_7.4 and K_v_7.5 subunits of the K_v_7 channel and reduces the channel activity, likely through PKC-dependent phosphorylation of the channel protein [259]. Thromboxane A2 also induces pulmonary artery contraction via a mechanism involving PKCζ activation and K_v_ channel inhibition [260]. In rabbit coronary artery VSMCs, Ang II and ET-1 inhibit K_v_ channel through the activation of PKCε and reduce K_IR_ channel activity through the activation of PKCα [261,262]. Vasoconstrictors also inhibit K_ATP_ activity through a PKC-dependent pathway [246,263], and direct stimulation of PKC by phorbol esters reduces K_ATP_ current in mesenteric artery VSMCs [264]. PKCδ, PKCθ, and PKCμ also play a role in VSM mechanotransduction and stretch-induced myogenic tone [265,266]. In VSMCs from rat cerebral artery, cell swelling, DAG and phorbol 12-myristate 13-acetate (PMA) activate stretch-sensitive cation current and TRP channels. Also, vascular myogenic tone partly involves the stimulation of PLC, hydrolysis of PIP_2_, increased production of DAG, and activation of PKC, which in turn activates L-type VDCCs, increases [Ca^2+^]_c,_ and promotes vasoconstriction [267,268]. PKC also phosphorylates and activates the Na^+^/H^+^ exchanger, leading to increased cytosolic pH, cell alkalinization, and vascular contraction [269,270,271,272]. Also, while PKC modulates endothelial eNOS activity, NO production, and vasodilation [273], it could inhibit guanylate cyclase in VSMCs, leading to inhibition of vasodilation and consequent increases in vasoconstriction [274].

PKC could also modulate vascular [Ca^2+^]_c_ and contraction in a feed-back fashion. In VSMCs, low DAG concentrations activate TRPC6 independent of PKC, but high DAG levels inhibit TRPC6 and SOCs activity via PKC- and Ca^2+^-dependent mechanisms [268,275]. In rabbit ear and mesenteric artery VSMCs, high Ang II concentrations and increased DAG production and PKC activity inhibit TRPCs [276]. PKC could also phosphorylate MLC and MLCK, thus antagonizing Ca^2+^-induced actin–myosin cross-bridge cycling and VSMC contraction [277]. In human VSMCs, PKC could also promote the release of vasodilator substances such as C-type natriuretic peptide (CNP) [278,279]. Platelet-derived growth factor (PDGF) and the phorbol ester PMA increase CNP expression in SMCs via PKCα- and PKCδ-dependent pathways [280]. PKC could also activate Ca^2+^ removal via PMCA or SERCA, leading to decreased VSMC [Ca^2+^]_c_ and contraction. In isolated cardiac SR fractions, PKC activates SERCA [281]. PKC also inhibits the α1 subunit of Na^+^/K^+^-ATPase, thus affecting the cell Na^+^ and K^+^ balance, membrane potential, and VDCC activity [282].

### 8.4. Ca^2+^ and Rho-Kinase (ROCK)

Vasoconstrictor agonists also activate GPCR and the small G-protein RhoA. RhoA binds GTP and activates Rho-associated coiled-coil protein kinase (Rho-kinase, ROCK). In the absence of substantial increases in [Ca^2+^]_c_, ROCK activation causes Ca^2+^ sensitization of the contractile proteins and enhancement of VSMC contraction [283,284,285,286].

Structurally, ROCK is composed of a kinase domain at the N-terminal, a coiled-coil region harboring the Rho-binding domain, followed by a pleckstrin-homology domain with a cysteine-rich region at the C-terminal that facilitates ROCK association to the surface membrane. ROCK isoforms include highly homologous ROCK-1 (ROCK-I, ROKβ) and ROCK-2 (ROCK-II, ROKα) [287,288,289,290,291]. Both ROCK-1 and ROCK-2 are ubiquitously expressed in multiple tissues, particularly VSMCs and the heart [292]. Chronic administration of Ang II in mice activates AT_1_R and upregulates ROCK in the coronary artery [293]. Also, interleukin-1β upregulates ROCK-1 and ROCK-2 expression, likely via PKC-mediated activation of nuclear factor kappa B (NFκB) [293].

In resting cells, RhoA is located in the cytoplasm bound to guanosine diphosphate (GDP), but upon vasoconstrictor–receptor interaction, it translocates to the surface membrane and exchanges GDP with GTP [286]. ROCKs also mainly reside in the cytoplasm and translocate to the surface membrane upon their binding to and activation by RhoA [288,289]. The interaction of activated RhoA with ROCK at its Rho-binding domain induces conformational changes that allow the interaction of the ROCK catalytic domain and associated Ser/Thr kinase activity with its substrates [294].

ROCK phosphorylates several substrates, particularly those involved in actin filament and cytoskeleton organization [295,296]. Other ROCK substrates include MYPT-1 [297], CPI-17 [298], MLC [299], and CaP [300]. ROCK causes Ca^2+^ sensitization by directly phosphorylating MYPT1, thus inhibiting MLC phosphatase and decreasing its binding to MLC. ROCK also phosphorylates CPI-17, which binds to and inhibits the MLC phosphatase catalytic subunit [301]. ROCK inhibition of MLC phosphatase enhances MLC phosphorylation, actin–myosin cross-bridge cycling, and VSMC contraction [302,303]. Vasoconstrictor agonists can also increase arachidonic acid by stimulating the hydrolysis of membrane phospholipids by PLA2 or transforming DAG by DAG lipase. Arachidonic acid binds to the pleckstrin-homology domain and regulatory region of ROCK, thus reversing their inhibitory effect on the catalytic domain, and enhancing the activity of ROCK independently of RhoA [304,305,306], leading to inhibition of MLC phosphatase, increased Ca^2+^-dependent MLC phosphorylation and enhanced VSMC contraction [307].

ROCK-mediated organization of the actin cytoskeleton is also involved in cell survival/apoptosis, proliferation, migration, and differentiation [308,309,310]. In addition to inhibiting MLC phosphatase [311], ROCK promotes LIM-kinase-2 activity, the formation of stress fibers, focal adhesions and membrane blebs [312], ezrin/radixin/moesin phosphorylation [313], and adducin phosphorylation and cell motility [314]. In contrast, ROCK-mediated phosphorylation of troponin reduces contraction of cardiac myocytes [315]. Other small G-proteins such as RhoE bind to ROCK1 N-terminus kinase domain, thus competing with RhoA binding and reducing ROCK activity [316].

## 9. Ca^2+^ and Extracellular Matrix (ECM)

Matrix metalloproteinases (MMPs) are a large family of Ca^2+^ and zinc-sensitive proteolytic enzymes that are involved in ECM remodeling, protein degradation, and turnover. MMPs are also involved in other physiological processes including embryonic development, angiogenesis, tissue repair, and modulation of vascular contraction/relaxation. Ca^2+^ is central to MMP activation, stability, and function, making it a crucial regulator of ECM integrity [317].

MMPs share a similar structure, comprising a pro-peptide domain, a catalytic domain, and a hemopexin-like C-terminus domain. Most MMPs are first synthesized as inactive zymogens or proenzymes, containing a pro-peptide domain that inhibits the protease activity by interacting with the catalytic zinc ion in the active site. The MMP catalytic domain contains binding sites for both Ca^2+^ and zinc. Ca^2+^ binding to the catalytic domain contributes to the structural stability of MMPs, prevents their premature denaturation, protects them from degradation, and maintains their readiness for activation [317,318]. Activation of MMPs involves the removal of the pro-peptide domain, a process regulated by various proteolytic enzymes and local Ca^2+^ concentrations. Extracellular signals, such as inflammatory cytokines and growth factors, trigger the activation of membrane-type MMPs (MT-MMPs), which cleave the pro-peptide domain of other MMPs, and expose the catalytic zinc ion in the active site.

Zinc regulates the catalytic activity of MMPs by coordinating water molecules and initiating nucleophilic attacks on peptide bonds in ECM proteins. The water molecule, bound to zinc, attacks the scissile bond of the substrate, leading to hydrolysis and breakdown of the protein structure. In addition to the catalytic role of zinc, Ca^2+^ enhances the efficiency and stability of the catalytic process. Ca^2+^ binding at auxiliary sites adjacent to the zinc-binding site supports the proper folding of MMP, optimizing the spatial orientation of the catalytic zinc, facilitating substrate binding, and allowing MMPs to function optimally in the ECM complex environment [318,319,320]. Ca^2+^ binding induces conformational changes in the catalytic domain that stabilize the active site, facilitating MMP access and affinity to ECM substrates like collagen, elastin, and fibronectin and allowing MMPs to degrade ECM components [317,318].

The activity of MMPs is tightly regulated to prevent excessive ECM degradation, primarily through tissue inhibitors of metalloproteinases (TIMPs), which also depend on Ca^2+^ for stability. TIMPs are endogenous inhibitors that bind to the active sites of MMPs, thus blocking their access to ECM substrates and preventing uncontrolled ECM degradation. The interaction between TIMPs and MMPs is stabilized by Ca^2+^, ensuring that MMP activity remains controlled in normal physiological conditions. Fluctuations in Ca^2+^ levels influence the binding affinity of TIMPs for MMPs. In environments with elevated Ca^2+^ levels, TIMPs dissociate more readily, allowing MMPs to become active. This dynamic regulation allows MMPs to be rapidly activated in response to tissue injury or inflammation while preventing prolonged ECM degradation under normal conditions [321,322].

## 10. Ca^2+^ Dysregulation in Vascular Disease

Vascular dysfunction, decreased endothelium-dependent vasodilation, increased vasoconstriction, excessive vascular remodeling, and increased vascular permeability could lead to major cardiovascular disorders such as HTN, age-related vascular stiffness, atherosclerosis, and CAD.

### 10.1. Dysregulated Ca^2+^ Signaling in Hypertension (HTN)

**Figure 5:**
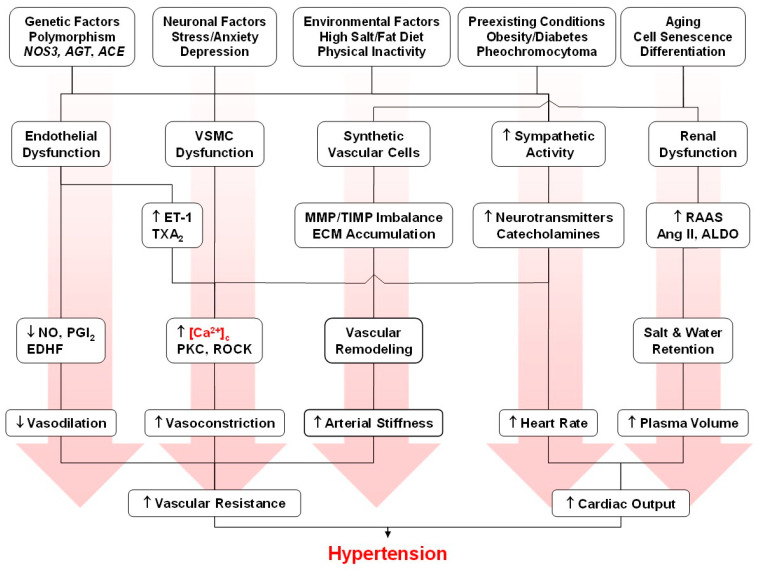
Pathophysiological mechanisms and dysregulated Ca^2+^ signaling dynamics in hypertension. Genetic, neuronal, and environmental factors, as well as preexisting conditions, cause EC and VSMC dysfunction and increased sympathetic activity, leading to changes in [Ca^2+^]_c_ and other signaling mechanisms and resulting in decreased vasodilation and increased vasoconstriction and vascular resistance. Increased sympathetic activity also increases heart rate. Aging and cell senescence cause differentiation of vascular cells into synthetic phenotype, increased vascular remodeling, and arterial stiffness, thus exacerbating vascular resistance. Aging also causes renal dysfunction and increases in the renin–angiotensin–aldosterone system (RAAS), Ang II, aldosterone (ALDO), sodium and water retention, and plasma volume, which together with the increased heart rate increase cardiac output. The increases in vascular resistance and cardiac output lead to elevation of blood pressure and hypertension.

Dysregulations of Ca^2+^ signaling dynamics have been implicated in EC dysfunction, VSMC hyperactivity, vascular remodeling, and the pathogenesis of various forms of HTN (Figure 5). ECs play a central role in BP regulation by releasing the vasodilatory factors NO and PGI_2_ through Ca^2+^-dependent pathways. In HTN, impaired EC Ca^2+^ signaling mechanisms and Ca^2+^ entry through TRPCs lead to decreased eNOS activity, deficient NO production, and reduced vasodilatory capacity, causing persistent vasoconstriction and increased vascular resistance. Also, in HTN increased oxidative stress and ROS disrupt EC Ca^2+^ homeostasis and Ca^2+^ channel function and impair eNOS activity and NO bioavailability, leading to reduced EC Ca^2+^ sensitivity and decreased vascular relaxation [323].

Ca^2+^ is a major determinant of VSMC contraction and maintained vascular tone. Abnormal Ca^2+^ handling mechanisms lead to increased [Ca^2+^]_c_, excessive vasoconstriction, increased vascular resistance, and HTN. In hypertensive individuals, L-type VDCCs show increased density and activity in VSMCs, resulting in exaggerated Ca^2+^ influx and sustained vasoconstriction [324]. HTN is often associated with hyperactivity of the sympathetic nervous system (SNS), which augments Ca^2+^-dependent vasoconstriction. Sympathetic nerve terminals release norepinephrine, which binds to α1-adrenergic receptors in VSMCs, activating GPCR and increasing [Ca^2+^]_c_, vasoconstriction, and BP [325,326]. SNS activation also stimulates the adrenal medulla cells, causing increases in [Ca^2+^]_c_ and the release of the catecholamines epinephrine and norepinephrine, which increase vascular tone, heart rate, and cardiac output, exacerbating HTN, particularly under conditions of chronic stress and sympathetic overdrive [327].

Excessive activation of PKC- and ROCK-dependent pathways also leads to Ca^2+^ sensitization of the contractile myofilaments, increased vascular tone and resistance, further contributing to elevated BP [21]. PKC plays a role in the pathogenesis of vascular stenosis and HTN [20,47], and PKC mutations could affect the susceptibility of an individual to increased vascular reactivity and HTN. PKCα plays a role in Ca^2+^-dependent VSMC contraction [328], and increased PKCα expression has been suggested as an underlying mechanism in the pathogenesis of HTN [329]. In normotensive rats, PKCα is located largely in the VSMC cytoplasm, but it shows hyperactivation and redistribution to the VSMC surface membrane in hypertensive rats [329]. PKCα, β, and δ also influence VSMC migration by enhancing actin polymerization and cell adhesion [330,331,332]. PKCα is implicated in ROS-induced VSMCs migration [333], and PKCε promotes VSMC migration by upregulating MMP-2 and MMP-9 [334,335,336]. PKC also contributes to VSMC proliferation. PKCβ mediates synergistic proliferative effects of PDGF and high glucose in human coronary VSMCs [337], and the PKCβ inhibitor LY-379196 attenuates cell growth [338]. In rats subjected to aortic balloon injury, PKCε activation by ψεRACK augments neointima formation, whereas PKCε inhibition by εV1-2 decreases PDGF-induced VSMC proliferation, migration and ERK phosphorylation, neointima development, and vessel lumen narrowing [339]. Also, repetitive PKC stimulation using phorbol myristate acetate (PMA) decreases PKCε expression and reduces VSMC proliferation [340].

ROCK has been implicated in the pathogenesis of cancer, metabolic and neurological disorders, and essential HTN [341,342,343]. Hypertensive animal models demonstrate increases in the activity of RhoA/ROCK [344], and ROCK inhibitors such as Y-27632 and fasudil reduce BP in experimental HTN [345]. ROCK inhibitors suppress the hypertrophic vascular media and the perivascular fibrosis observed in coronary arteries of the spontaneously hypertensive rat (SHR) [346] and in the chronic NOS inhibition rat model of HTN, which show increased RhoA/ROCK activity [347]. AT_1_R inhibition also reduces RhoA/ROCK activity in hypertensive rat models, indicating that ROCK activation results from enhanced Ang II/AT_1_R activity [347]. Also, chronic Ang II infusion in rats augments coronary arterial RhoA/ROCK activity, tunica media thickness, and perivascular fibrosis, and ROCK inhibitors attenuate Ang II-induced coronary hypertrophy/fibrosis [348], superoxide anion production [348], and monocyte chemoattractant protein-1 and PAI-1 levels [349,350]. Importantly, HTN increases mechanical strain on the vascular wall and promotes VSMC proliferation [351], and ROCK inhibitors reduce stretch-induced MAPK activation and VSMC growth/proliferation [352,353].

Dysregulated MMP activity can lead to aberrant ECM degradation and pathological conditions such as cancer, arthritis, and cardiovascular disease. Changes in plasma and vascular tissue MMP-2 have been associated with increased vascular remodeling and HTN [354]. Imbalance in vascular MMP activity and vasoconstriction/vasodilation mechanisms could also promote VSMC switching from contractile to synthetic phenotype, enhancing MMP-2 release and facilitating cell growth, migration, deposition of ECM components, and exacerbating vascular remodeling and HTN [355].

Ca^2+^ signaling in the renal tubular cells plays a critical role in the regulation of sodium reabsorption, fluid balance, plasma volume and BP. The renin–angiotensin–aldosterone system (RAAS) controls BP through the regulation of renal tubular Ca^2+^ dynamics. Ang II binding to AT_1_R in renal tubular cells stimulates Ca^2+^ mobilization from the intracellular stores and Ca^2+^ influx and promotes sodium retention and volume expansion. In the nephron distal convoluted tubule and collecting duct, Ca^2+^ modulates the activity of the epithelial sodium channel (ENaC) and the sodium–chloride cotransporter (NCC). In HTN, increased RAAS activity and Ang II elevate [Ca^2+^]_c_ in renal tubular cells and upregulate sodium transporters, thereby increasing sodium reabsorption and extracellular fluid and plasma volume [356]. This Ca^2+^-dependent RAAS pathway creates a positive feed-forward loop, whereby elevated Ang II levels maintain high renal tubular [Ca^2+^]_c_, exacerbating sodium retention and HTN [357].

### 10.2. Ca^2+^ Dysregulation in HTN-in-Pregnancy (HTN-Preg) and Preeclampsia

HTN-Preg is a complication of pregnancy with marked maternal and fetal morbidity and mortality. HTN-Preg encompasses preexisting HTN that precedes pregnancy, preeclampsia (PE)/eclampsia, HTN with superimposed PE, and gestational HTN. A common abnormality in these forms of HTN-Preg is dysregulation of Ca^2+^ signaling dynamics, which disrupts EC function, vascular tone, and renal sodium handling.

Endothelial dysfunction is a hallmark of HTN-Preg with impaired Ca^2+^-dependent production of NO and PGI_2_ and decreased vasodilatory responses [358]. HTN-Preg is also associated with oxidative stress and increased ROS, which decrease NO bioavailability and lead to reduced vasodilation, angiogenesis and placental perfusion, and increased placental ischemia. Elevated ROS levels in the PE placenta disrupt Ca^2+^ signaling pathways, leading to Ca^2+^ overload in trophoblast cells and impairment of their function, further exacerbating placental insufficiency and causing adverse pregnancy outcomes, fetal growth restriction, and preterm birth [358,359]. Piezo1 and TRPV4 are co-regulated mechanosensors in ECs, and both channels could disrupt Ca^2+^ homeostasis and mediate aberrant placental endothelial function, placental development, and vascularization in early onset PE [360]. Abnormal Ca^2+^ handling in the spiral arteries and subsequent placental ischemia could also lead to an imbalance in angiogenic and anti-angiogenic factors and increased soluble fms-like tyrosine kinase-1 (sFlt-1), which counteracts placental growth factor and vascular endothelial growth factor (VEGF), further reducing placental perfusion and causing systemic vascular dysfunction [361].

In HTN-Preg, increased Ca^2+^ channel activity, Ca^2+^ influx, and Ca^2+^ sensitivity in VSMCs lead to sustained vasoconstriction, increased vascular resistance, and impaired blood flow. Increased vasoconstriction in uterine and placental vessels compromises uteroplacental blood flow and leads to fetal growth restriction [362,363]. Additionally, the RhoA/ROCK pathway inhibits MLC phosphatase and promotes Ca^2+^ sensitization in VSMCs. In HTN-Preg, ROCK is highly active, leading to sustained myosin phosphorylation and prolonged vascular contraction even at low Ca^2+^ levels, resulting in increased vascular tone and BP [364].

Several studies support a role of placental hypoxia/ischemia as an early event in the pathogenesis of PE. Hypoxia attenuates the vasodilator effects of sex hormones and enhances PKC activity and vascular tone in uterine arteries of pregnant sheep [365]. During pregnancy, increased BK_Ca_ channel activity inhibits PKC-mediated contraction in ovine uterine arteries, and gestational hypoxia upregulates PKC and inhibits BK_Ca_ [366]. Hypoxia can also inhibit K_IR_ channel activity through a PKC-mediated pathway, further contributing to the maladaptation of uterine vascular function observed in PE [247]. Rat cardiac myocytes treated with IgG from women with PE show augmented AT_1_R-induced response, which is attenuated by PKC inhibitors such as calphostin C, lending support to a PKC role in PE [367].

Dysregulated RAAS and renal Ca^2+^ signaling also play a role in the increased renal sodium reabsorption, volume expansion, and elevated BP in HTN-Preg and PE. Pro-renin, angiotensinogen, angiotensin converting enzyme (ACE), Ang I, and Ang II have been identified in the placenta [368]. Other Ang II-related biomolecular mechanisms, including AT_1_R and bradykinin B2 receptor heterodimerization and increased circulating AT_1_R agonistic antibodies, have been proposed in PE [369]. Also, our laboratory has shown enhanced maintained [Ca^2+^]_c_ and contraction induced by Ang II, KCl, and BAY K-8644 in renal arterial VSMCs from a rat model of placental ischemia, suggesting enhanced Ca^2+^ entry mechanisms in small renal arteries as a major factor in the increased renal arterial resistance during HTN-Preg [370].

### 10.3. Ca^2+^ Dysregulation in Pulmonary Arterial Hypertension (PAH)

PAH is a severe and progressive cardiovascular disorder involving persistent vasoconstriction and excessive remodeling of the pulmonary arterioles, leading to severe narrowing of the pulmonary vessels, increased pulmonary arterial resistance, right ventricular failure, and ultimately death. Dysregulated Ca^2+^ signaling dynamics play a role in the pathogenesis of PAH, contributing to sustained pulmonary vasoconstriction, vascular remodeling, and proliferation of pulmonary arterial SMCs (PASMCs).

ECs play a major role in regulating pulmonary artery tone by releasing vasodilators such as NO and PGI_2_ in response to increases in [Ca^2+^]_c_. In PAH, endothelial dysfunction and compromised Ca^2+^ signaling decrease eNOS activity and NO production, thus reducing NO-mediated vasodilation and contributing to sustained vasoconstriction [371]. PAH-associated endothelial dysfunction also impairs Ca^2+^-dependent PGI_2_ production, thus reducing an important counter-regulatory mechanism to vasoconstriction and causing further elevation in pulmonary arterial pressure [372].

A hallmark of PAH is sustained pulmonary vasoconstriction, driven by elevated [Ca^2+^]_c_ and increased Ca^2+^ sensitivity in PASMCs. PASMCs from PAH patients show increased expression/activity of L-type VDCCs, leading to increased Ca^2+^ influx, prolonged vasoconstriction and increased pulmonary arterial pressure [373]. Also, Piezo1 expression and [Ca^2+^]_c_ are increased in PASMCs [374] and pulmonary arterial ECs from idiopathic PAH patients and in the pulmonary artery and PASMCs from animal models of severe PAH, suggesting that excessive Ca^2+^ entry through mechanosensitive stretch-activated channels plays a critical role in PAH-related increases in pulmonary vasoconstriction and remodeling [375]. Studies examined the role of the spatial organization between surface membrane stretch-activated channels and intracellular Ca^2+^ stores in SR, mitochondria, and lysosomes in the response to stretch in freshly isolated PASMCs from control and chronically hypoxic and monocrotaline-treated rat models of PAH. Co-immunolabelling experiments demonstrated different subcellular segregation between the SR ryanodine receptors RyR1, RyR2, and RyR3, the SERCA2 pumps SERCA2a and SERCA2b, mitochondria, and lysosomes in control versus PAH PASMCs. Stretching the membrane of control PASMCs activated Ca^2+^ influx through stretch-activated channels, which was amplified by cell hyperpolarization and Ca^2+^ release from subplasmalemmal RyR1 and was then buffered by mitochondria. This Ca^2+^ response to stretch was enhanced in PAH PASMCs due to the hyper-reactivity of stretch-activated channels and a greater Ca^2+^ amplification by all RyR subtypes. These observations suggest an important role of spatial organization of RyRs and Ca^2+^ stores in PASMC cell signaling that could be altered due to hyper-reactive stretch-activated channels in PAH [376]. Ca^2+^ sensitization pathways in PASMCs also increase the sensitivity of the contractile machinery to [Ca^2+^]_c_ and further amplify pulmonary vasoconstriction. Activation of RhoA/ROCK inhibits MLC phosphatase and maintains MLC phosphorylation at low [Ca^2+^]_c_, thus promoting Ca^2+^ sensitization, sustained vasoconstriction, increased pulmonary vascular resistance, and PAH [377]. Ca^2+^ signaling and prolonged Ca^2+^ influx also promote PASMC proliferation and migration, contributing to pulmonary vascular remodeling and thickening of the arterial wall [378]. Elevated [Ca^2+^]_c_ in PASMCs also activates transcription factors including NFAT and hypoxia-inducible factor-1α, which drive genes associated with cell proliferation and survival. For instance, sustained Ca^2+^ influx promotes NFAT translocation to the nucleus, where it upregulates growth-promoting genes, thus contributing to excessive PASMC proliferation [379,380].

In PAH, mitochondrial Ca^2+^ dysregulation and increased Ca^2+^ uptake disrupts energy production, leading to metabolic shifts in PASMCs. Mitochondrial Ca^2+^ overload impairs oxidative phosphorylation and promotes a shift to glycolysis, even under normoxic conditions (Warburg effect), to support the bioenergetic and biosynthetic needs of proliferating PASMCs, thus fueling vascular remodeling [380,381]. Mitochondrial Ca^2+^ dysregulation also increases the generation of ROS, which exacerbate cellular oxidative stress and affect Ca^2+^ channels and transporters, further disrupting Ca^2+^ homeostasis and enhancing the pro-proliferative environment for PASMCs in PAH [382].

Sleep apnea could cause systemic HTN and PAH [383,384]. Studies in a rat model of sleep apnea induced by intermittent exposure to eucapnic hypoxia showed ET-1-dependent systemic HTN, elevated circulating levels of ET-1, and increased vasoconstriction in mesenteric vessels, possibly through PKCδ-mediated increases in Ca^2+^ sensitivity of the contractile proteins [385]. Intermittent hypoxia also activates PKCβ-mediated increases in the vasoreactivity of pulmonary arteries to ET-1 and other vasoconstrictors [384,386]. In fawnhooded pulmonary hypertensive rats, PKC likely inhibits BK_Ca_ channel activity, leading to membrane depolarization, activation of VDCCs, and increased pulmonary vasoconstriction [387]. Pulmonary arteries of newborn swine exposed to hypoxia also show PKC-mediated sensitization of contractile proteins to [Ca^2+^]_c_ through the increased phosphorylation of CPI-17 and the inhibition of MLC phosphatase [388].

RhoA and ROCK also play key roles in the excessive vasoconstriction and vascular remodeling in PAH [389,390]. ROCK participates in the hypoxia-induced downregulation of eNOS expression and NO production in human pulmonary ECs [391]. Chronic hypoxia in rats is also associated with increased ROCK levels and ROCK-mediated Ca^2+^ sensitivity of contractile myofilaments in pulmonary arterioles [392]. ROCK inhibition by Y-27632 attenuates hypoxia-induced pulmonary vasoconstriction, remodeling, and PAH [393]. Also, the ROCK inhibitor fasudil, administered either orally or by inhalation, reduces monocrotaline-induced PAH in rats by improving endothelium-dependent pulmonary artery relaxation and inhibiting VSMC proliferation, macrophage infiltration, and pulmonary fibrosis [342,390,394,395].

### 10.4. Ca^2+^ Dysregulation in Age-Related Cell Senescence and Arterial Stiffness

Aging is a primary risk factor for cardiovascular disease, with older individuals having a higher incidence of HTN, atherosclerosis, and heart failure. Age-associated changes in Ca^2+^ signaling contribute to cardiovascular dysfunction, affecting ECs, VSMCs, cardiomyocytes, and immune cells. Dysregulated Ca^2+^ homeostasis exacerbates endothelial dysfunction, cell senescence, and arterial stiffness [396].

Aging and senescent ECs show impaired Ca^2+^ signaling, reduced Ca^2+^ influx via TRP channels and reduced vasodilator factors such as NO and PGI_2_, leading to increased vascular tone, and age-associated HTN [397,398]. Age-related oxidative stress also interferes with Ca^2+^ channels and pump activity and in turn exacerbates Ca^2+^ dysregulation. Excessive ROS alter TRP channel function and reduce the activity of SERCA, limiting Ca^2+^ reuptake into the ER and leading to intracellular Ca^2+^ overload, thus compromising EC function, diminishing vasodilatory capacity, and increasing vascular resistance [399].

Arterial stiffness is a hallmark of aging, contributing to HTN and increased cardiovascular risk. In aging arteries, VSMCs exhibit increased expression of L-type VDCCs, leading to enhanced Ca^2+^ influx, sustained VSMC contraction, increased vascular tone, and decreased arterial compliance. Also, Ca^2+^ sensitization through the ROCK pathway enhances contractile force in aging VSMCs, contributing to vascular stiffness and elevated systolic BP in older individuals [400,401]. Dysregulated Ca^2+^ signaling and increased [Ca^2+^]_c_ in aging VSMCs also activate MMPs, which alter the collagen/elastin ratio, reduce arterial elasticity, and promote age-related vascular remodeling, arterial stiffening, and HTN [402,403].

In the aging heart, Ca^2+^ dysregulation in cardiomyocytes contributes to diastolic dysfunction and increased susceptibility to heart failure. SERCA function declines with age, reducing Ca^2+^ reuptake into the SR and prolonging elevated [Ca^2+^]_c_ during diastole, thus disrupting the timing of cardiac relaxation, causing diastolic dysfunction and increasing the risk of age-related heart failure with preserved ejection fraction (HFpEF) [404]. Also, in aging cardiomyocytes, Ca^2+^ leakage from SR through RyRs exacerbates [Ca^2+^]_c_ overload, contributing to contractile dysfunction and arrhythmogenesis [405,406]. Mitochondrial Ca^2+^ handling is also disrupted in aging cardiomyocytes, leading to mitochondrial Ca^2+^ overload, metabolic dysfunction, impaired ATP synthesis, oxidative stress, and increased ROS, which damage mitochondrial and cytosolic proteins, further disrupting Ca^2+^ homeostasis and contributing to age-related decline in cardiac output and increased risk of heart failure [404].

Dysregulated Ca^2+^ signaling also causes cellular senescence and irreversible cell cycle arrest. In aging cells, elevated [Ca^2+^]_c_ promotes the development of a senescence-associated secretory phenotype (SASP), which shows increases in the release of inflammatory cytokines, chemokines, and growth factors. Ca^2+^-dependent activation of NF-κB upregulates SASP components, leading to chronic cardiovascular inflammation and accelerated age-related vascular and cardiac dysfunction [407,408]. Persistent elevation of [Ca^2+^]_c_ has also been linked to DNA damage, and senescence of vascular and myocardial cells [409]. Increased [Ca^2+^]_c_ activates calpains, a family of Ca^2+^-dependent proteases that degrade nuclear proteins involved in DNA repair, thus increasing the number of senescent vascular cells which release inflammatory mediators and further exacerbate tissue aging and dysfunction [410,411].

### 10.5. Ca^2+^ Dysregulation in Vascular Inflammation, Atherosclerosis, and Calcification

**Figure 6:**
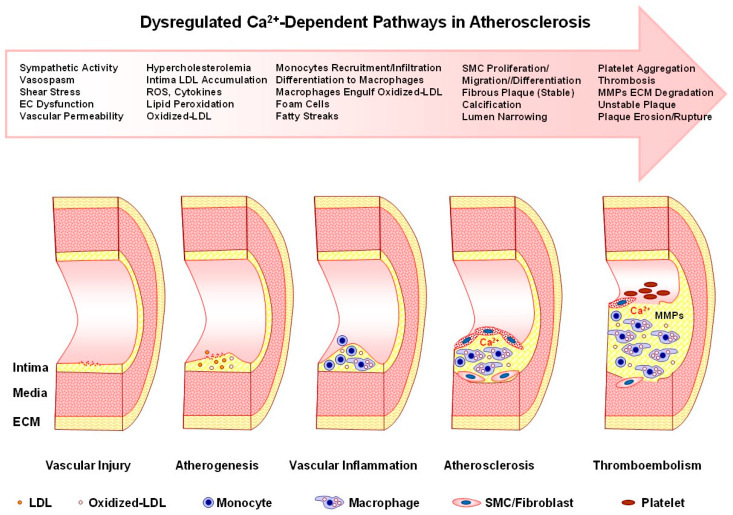
Dysregulated Ca^2+^ signaling dynamics, vascular inflammation, and calcification in atherosclerosis. HTN and high plasma low-density lipoprotein (LDL) cholesterol cause endothelium injury and accumulation of oxidized-LDL in the vascular intima. Dysregulated Ca^2+^ signaling in ECs also triggers inflammatory responses and recruitment/infiltration of monocytes to the injury site. Monocytes differentiate into macrophages, which engulf oxidized-LDL and become foam cells, thus creating a fatty streak and atherosclerotic plaque. Inflammatory cytokines promote VSMC proliferation, migration, and differentiation into fibroblasts, covering the fatty streak and forming a stable, fibrous plaque. ROS and inflammatory cytokines promote VSMC phenotypic switching to an osteogenic-like phenotype and deposition of calcium phosphate crystals, initiating vascular plaque calcification. Progressive plaque growth, blood vessel narrowing, and platelet aggregation promote thrombosis and vascular blockage. Activation of Ca^2+^-dependent MMPs causes vascular remodeling, plaque instability and rupture, and dislodgment of thrombi into the circulation, causing serious thromboembolism and cardiovascular events.

The endothelium represents an important line of defense to combat vascular inflammation and atherosclerosis. HTN and high plasma low-density lipoprotein (LDL) cholesterol cause endothelium injury and the accumulation of oxidized-LDL in the vascular intima (Figure 6). Dysregulated Ca^2+^ signaling in ECs also triggers inflammatory responses within the vascular wall and promotes atherogenesis and recruitment/infiltration of inflammatory cells. Upon exposure to oxidized-LDL ECs respond by elevating [Ca^2+^]_c_, which activates NF-κB and activator protein-1 (AP-1) and in turn increases the expression of intercellular adhesion molecule-1 (ICAM-1) and vascular cell adhesion molecule-1 (VCAM-1), facilitating the adhesion, recruitment, and infiltration of leukocytes and monocytes to the injury site in the vascular wall [412,413]. Monocytes that infiltrate the arterial intima differentiate into macrophages, which engulf oxidized-LDL and become foam cells, thus creating a fatty streak and atherosclerotic plaque. Differentiated macrophages exposed to oxidized-LDL show a sustained increase in store-operated Ca^2+^ entry and [Ca^2+^]_c_, which activates NF-κB and MAPK, leading to the release of pro-inflammatory cytokines such as interleukin-1β (IL-1β) and tumor necrosis factor-α (TNF-α) and the nucleotide-binding oligomerization domain (NOD)-like receptor family pyrin domain containing-3 (NLRP3) inflammasome, which exacerbate plaque inflammation and promote VSMC proliferation [412,413,414]. VSMCs migrate and differentiate into fibroblast-like cells, covering the fatty streak and forming a stable, fibrous plaque. High [Ca^2+^]_c_ also promotes cholesterol ester accumulation within the macrophages by enhancing the activity of the enzymes involved in cholesterol metabolism. Foam cells continue to release inflammatory cytokines, causing the further recruitment of immune cells and perpetuation of an inflammatory microenvironment that contributes to plaque growth and necrosis, creating a core that is prone to destabilization and rupture [415,416,417,418].

Vascular calcification and Ca^2+^ deposition in the arterial wall are prominent features of advanced atherosclerotic plaques and could cause plaque rupture and adverse cardiovascular events. Ca^2+^ plays a critical role in osteogenic transformation of VSMCs and vascular calcification. Oxidative stress and inflammatory cytokines promote VSMCs phenotypic switching from a contractile to an osteogenic-like phenotype. Increased [Ca^2+^]_c_ activates runt-related transcription factor 2 (RUNX2), an important transcription factor involved in bone formation, and promotes the expression of osteogenic markers such as alkaline phosphatase and bone morphogenetic proteins (BMPs) [419,420]. This Ca^2+^-induced transformation drives VSMCs to deposit calcium phosphate crystals, initiating vascular plaque calcification. Calcifying VSMCs also release Ca^2+^-rich matrix vesicles, which function as nucleation sites for hydroxyapatite formation, the primary mineral component in bone. These matrix vesicles aggregate within the plaque, forming microcalcifications that stiffen the arterial wall and increase the risk of plaque rupture. High [Ca^2+^]_c_ stimulates vesicle release, exacerbating calcification and promoting atherosclerotic plaque progression [421,422]. Progressive plaque growth, blood vessel narrowing, and platelet aggregation promote thrombosis and vascular occlusion. Activation of Ca^2+^-dependent MMPs causes vascular remodeling, plaque instability and rupture, and dislodgment of thrombi into the circulation, causing serious thromboembolism and cardiovascular events.

### 10.6. Ca^2+^ Dysregulation in Coronary Artery Disease

Coronary artery disease (CAD) primarily results from vasospasm and atherosclerosis of the coronary arteries and is the leading cause of mortality globally. Dysregulated Ca^2+^ signaling in ECs, VSMCs, and immune cells plays a major role in CAD pathogenesis by promoting endothelial dysfunction, vasoconstriction, inflammation, and arterial calcification.

Dysregulation of EC Ca^2+^ signaling in CAD is associated with decreased NO bioavailability, increased oxidative stress, and accelerated atherosclerotic plaque formation. In CAD, impaired Ca^2+^ entry through TRPCs reduces eNOS activity and NO production in ECs, diminishing endothelium-dependent vasodilation and promoting inflammation and thrombosis. Elevated levels of ROS cause oxidative damage to TRPCs and Ca^2+^ pumps, exacerbate Ca^2+^ dysregulation, and decrease the Ca^2+^ sensitivity of ECs, limiting their ability to respond to vasodilatory stimuli. ROS-mediated Ca^2+^ dysregulation also promotes a pro-inflammatory environment that increases the adhesion of monocytes and their transformation into macrophages within the arterial wall and accelerates atherogenesis [423].

Dysregulated Ca^2+^ signaling in VSMCs promotes coronary vasospasm. In CAD, VSMC L-type VDCCs are overexpressed and hyperactive, leading to augmented Ca^2+^ influx, increased coronary vasoconstriction, reduced blood flow to the myocardium, acute oxygen supply–demand mismatch, and exacerbation of ischemic events. Also, in CAD, elevated activity of the RhoA/ROCK pathway and Ca^2+^ sensitization of the contractile proteins leads to persistent coronary vasoconstriction, which further precipitates ischemic events [424]

Dysregulated Ca^2+^ signaling also modulates immune cell infiltration and activation and promotes the inflammatory environment conducive for the formation of atherosclerotic plaques in CAD. In advanced CAD, persistent vascular inflammation exacerbates atherosclerosis progression, vascular calcification and thrombosis [414]. Activation of Ca^2+^-dependent MMPs also promotes vascular remodeling and plaque erosion, instability, and rupture and increases the risk of adverse cardiovascular events, including myocardial ischemia and infarction.

## 11. Restoration of Ca^2+^ Signaling Dynamics in Treatment of Vascular Disease

Several lines of treatment are currently available to improve Ca^2+^ signaling dynamics and manage HTN and CAD. Drugs that enhance endothelium-derived relaxing factors such as NO donors, soluble guanylyl cyclase (sGC) stimulators and phosphodiesterase type 5 (PDE5) inhibitors increase cGMP levels in VSMCs, leading to a decrease in [Ca^2+^]_c_, reduced vasoconstriction, and enhanced vasodilation [425]. β-Adrenergic receptor blockers such as propranolol decrease the heart workload. α-Methyldopa acts through a central mechanism to deplete norepinephrine and reduce sympathetic nerve activity. RAAS and Ang II/AT_1_R inhibitors decrease sodium and water retention and improve renal function. Diuretics decrease plasma volume and cardiac workload. For the management of PAH, sildenafil is used to inhibit PDE5 and prolong cGMP-mediated vasodilation in combination with PGI_2_ to enhance pulmonary arterial dilation and ET_A_R antagonists to decrease vasoconstriction. For CAD, nitroglycerins are used as NO donors to promote cGMP/PKG-mediated coronary artery dilation and alleviate ischemia. New approaches are also being developed for management of HTN. Aldosterone synthase, soluble epoxide hydrolase and vasopeptidase inhibitors, natriuretic peptide-A and vasoactive intestinal peptide receptor-2 agonists, and new mineralocorticoid receptor antagonists are being evaluated in clinical trials. Aminopeptidase-A, dopamine β-hydroxylase and Na^+^/H^+^ exchanger-3 inhibitors, ACE2, Ang-(1-7) and MasR agonists, and Ang II/AT_1_R vaccines are being assessed in phase I preclinical studies [426], and the effects of these agents on Ca^2+^ handling mechanisms need to be examined.

Many therapeutic strategies involve reducing VSMC [Ca^2+^]_c_ by inhibiting Ca^2+^ mobilization pathways, enhancing Ca^2+^ removal mechanisms, or inhibiting Ca^2+^-dependent enzymes/processes in order to restore normal vascular tone. Ca^2+^ channel blockers, such as amlodipine and nifedipine, reduce Ca^2+^ influx into VSMCs, promote vasodilation, and lower BP. Targeting PKC and RhoA/ROCK can reduce Ca^2+^ sensitization of the contractile proteins, VSMC contraction, and vascular resistance and alleviate HTN and arterial stiffness.

PKC inhibitors such as staurosporine and chelerythrine are nonspecific, as they compete with ATP in the catalytic domain and inhibit other ATP-binding protein kinases [427,428,429]. PKC inhibitors such as calphostin C compete with DAG at the DAG/phorbol ester binding site in the PKC regulatory domain and could be more specific [427]. Synthetic peptides mimicking the PKC pseudosubstrate sequence could be specific and efficient because they do not interfere with ATP binding and provide several points for interaction with the PKC molecule [430,431,432,433]. Also, siRNA for specific PKCs can be more isoform-specific with fewer side effects.

ROCK inhibitors also restore MLC phosphatase activity, reduce Ca^2+^ sensitization, and offer potential benefits in reducing BP and vascular remodeling [434]. Y-27632 lowers BP in animal models of HTN but has low specificity and safety profile because it competes with ATP at the kinase catalytic site for ROCK and other kinases [345]. In rat aorta and mesenteric artery, Y-27632 inhibits PKC-mediated vasoconstriction, suggesting either a downstream role of ROCK in the PKC pathway or off-target inhibition of PKC by Y-27632 [435]. Fasudil and its metabolite hyroxyfasudil are potent ROCK inhibitors with good safety profile and effectiveness in reducing coronary vasospasm in porcine models [436], and therefore have been evaluated in small-scale clinical trials [437,438,439] and approved for treatment of cerebral vasospasm following subarachnoid hemorrhage in Japan. However, fasudil competes with ATP at the kinase active site, and inhibits PKA with equal potency, which could interfere with its vasodilator action. Amino acid sequencing has shown differences between ROCK and PKA, which can be used to enhance the specificity of ROCK inhibitors [440].

MMPs are Ca^2+^ dependent proteolytic enzymes that promote ECM protein degradation and vascular remodeling in HTN. Doxycycline inhibits MMP-2 and MMP-9 [441] and reduces BP in animal models of HTN [354]. However, MMP inhibitors could cause severe musculoskeletal inflammation, tendinitis, joint pain, and stiffness [442,443], and doxycycline is the only MMP inhibitor approved by the Food and Drug Administration. Newly synthesized and biological MMP inhibitors with greater specificity and fewer side effects could reduce excessive tissue remodeling and help in the treatment of MMP-related vascular disease.

## 12. Discussion and Perspective

[Ca^2+^]_c_ is a major determining factor of the function of different vascular cells and is regulated by multiple Ca^2+^ channels, active pumps, and transport mechanisms in the surface membrane and specific intracellular organelles as well as Ca^2+^-sensitization mechanisms and regulatory proteins [444]. A critical balance between Ca^2+^ mobilization and Ca^2+^ removal pathways maintains resting [Ca^2+^]_c_ at constant levels. The elaborate Ca^2+^ signaling dynamics of each individual Ca^2+^ handling pathway and the interaction between different pathways protects vascular cells from excessive extracellular Ca^2+^ influx, maintains [Ca^2+^]_c_ under resting conditions, and allows sufficient elevation of [Ca^2+^]_c_ for the cells to perform their function. These elaborate Ca^2+^ signaling dynamics could involve multifunctional Ca^2+^ handling by the cell surface membrane and various organelles as well as regenerative, capacitative, cooperative, bidirectional, and reciprocal feed-forward and feed-back pathways. Normal Ca^2+^ signaling dynamics are fundamental for regulating cardiovascular cell function and mediating key processes such as vascular cell growth and proliferation, endothelium-dependent vascular relaxation, VSMC contraction, ECM deposition, and vascular remodeling [445]. Regulation of VSMC contraction involves a complex interplay of Ca^2+^ signaling, MLCK activation, and further modulation by PKC and RhoA/ROCK-dependent Ca^2+^ sensitization pathways [446,447]. Cyclic nucleotides including cAMP and cGMP activate PKA and PKG, respectively, and in turn influence VSMC Ca^2+^ handling mechanisms, [Ca^2+^]_c_, membrane potential, and Ca^2+^ sensitivity of the contractile machinery, causing opposing effects that inhibit VSMC contraction [448,449]. Dysregulation of these signaling pathways alters Ca^2+^ homeostasis and disrupts the critical balance between Ca^2+^ mobilization and Ca^2+^ removal pathways, resulting in prolonged elevations in [Ca^2+^]_c_ and cardiovascular diseases such as HTN, vascular inflammation, atherosclerosis, calcification, CAD, and heart failure [324,450,451,452]. Understanding the complex mechanisms regulating Ca^2+^ dynamics in vascular cells would help in identifying specific targets and developing targeted therapeutic strategies to restore normal Ca^2+^ signaling and vascular function.

Measurements of [Ca^2+^]_c_ provide an important molecular approach to detect changes in Ca^2+^ signaling dynamics in vascular cells. Ca^2+^ channel permeability and [Ca^2+^]_c_ are augmented in VSMCs isolated from animal models of HTN and coronary arterial vasospasm [13,324,370,450,451,453], and Ca^2+^ channel antagonists could ameliorate these vascular disorders [454]. HTN, coronary and cerebral vasospasm, and certain forms of CAD that do not respond adequately to Ca^2+^ antagonists may be more sensitive to other treatment modalities that target Ca^2+^ sensitization pathways, including PKC and ROCK [20,47,302,455,456]. The subcellular distribution of PKC and ROCK could determine their activity level and their contribution to vascular dysfunction and thereby help in the diagnosis, prognosis, and treatment of HTN [20,47]. Isoform-specific PKC inhibitors, pseudosubstrate inhibitory peptides, and siRNA could represent alternative therapies for Ca^2+^ antagonist-resistant forms of HTN [20,47]. Gene silencing of PKCδ utilizing short hairpin RNA (shRNA)-plasmid intravenous delivery improved vascular function and reduced BP in SHR [253]. Targeted-delivery of PKCβII and PKCδ antagonists using coated stents or balloons was efficient in reducing vasospasm in animal models [338]. ROCK inhibition using fasudil showed encouraging results in PAH and cerebral vasospasm. Specific modulators of MMP activity could maintain ECM structural integrity and improve vascular remodeling and arterial stiffness.

## 13. Conclusions

Ca^2+^ is a critical second messenger in many cell types, including vascular cells. Ca^2+^ signaling is a complex and intricate process involving several intracellular proteins and multiple channels and pumps working together to regulate vascular function. Significant progress has been made in characterizing the individual Ca^2+^ regulatory mechanisms, and advanced research points to interaction between the different pathways to achieve stable [Ca^2+^]_c_ in resting cells and threshold elevation in Ca^2+^ levels sufficient for cell activation, while avoiding an excessive and dangerous Ca^2+^ overload that could cause vascular cell death or dysfunction and vascular disease. Further in-depth understanding of the Ca^2+^ signaling dynamics holds the promise for identifying new diagnostic tools, advancing precision medicine, and improving clinical outcomes for patients with cardiovascular disease.

## Data Availability

Not applicable.

## Abbreviations

Ang II	angiotensin II
BK_Ca_	large conductance Ca^2+^-activated K^+^ channel
[Ca^2+^]_c_	cytosolic free Ca^2+^ concentration
CaD	caldesmon
CAD	coronary artery disease
CaM	calmodulin
cAMP	cyclic adenosine monophosphate
CaP	calponin
cGMP	cyclic guanosine monophosphate
CICR	Ca^2+^-induced Ca^2+^ release
CPI-17	PKC-potentiated phosphatase inhibitor protein-17
DAG	diacylglycerol
EC	endothelial cell
ECM	extracellular matrix
ER	endoplasmic reticulum
ERK	extracellular signal-regulated kinase
ET-1	endothelin-1
GPCR	G-protein-coupled receptor
HTN	hypertension
IP_3_	inositol 1,4,5-trisphosphate
K_v_	voltage-gated K^+^ channel
LTCC	L-type Ca_V_1.2 channel
MAPK	mitogen-activated protein kinase
MARCKS	myristoylated alanine-rich C kinase substrate
MLC	myosin light chain
MLCK	MLC kinase
NFAT	nuclear factor of activated T cells
NFκB	nuclear factor kappa B
NCX	Na^+^/Ca^2+^ exchanger
PDBu	phorbol 12,13-dibutyrate
PDGF	platelet-derived growth factor
PE	preeclampsia
PKA	cAMP-dependent protein kinase
PKC	protein kinase C
PKG	cGMP-dependent protein kinase
PMA	phorbol 12-myristate 13-acetate
PMCA	plasmalemmal Ca^2+^-ATPase
PLC	phospholipase C
PS	phosphatidylserine
RAAS	renin–angiotensin–aldosterone system
ROCK	Rho-kinase
ROS	reactive oxygen species
SERCA	sarco(endo)plasmic reticulum Ca^2+^-ATPase
SOC	store-operated Ca^2+^ channel
SR	sarcoplasmic reticulum
STIM1	stromal-interacting molecule 1
TRP	transient receptor potential
TTCC	T-type Ca_V_3.1/3.2/3.3
VDCC	voltage-dependent Ca^2+^ channel
VSMC	vascular smooth muscle cell
